# Speciation and population divergence in a mutualistic seed dispersing bird

**DOI:** 10.1038/s42003-022-03364-2

**Published:** 2022-05-09

**Authors:** Jordi de Raad, Martin Päckert, Martin Irestedt, Axel Janke, Alexey P. Kryukov, Jochen Martens, Yaroslav A. Red’kin, Yuehua Sun, Till Töpfer, Matthias Schleuning, Eike Lena Neuschulz, Maria A. Nilsson

**Affiliations:** 1 Senckenberg Biodiversity and Climate Research Centre (SBiK-F, Senckenberganlage 25, 60325 Frankfurt am Main, Germany; 2 LOEWE Centre for Translational Biodiversity Genomics (LOEWE-TBG), Senckenberganlage 25, 60325 Frankfurt, Germany; 3 Institute for Ecology, Evolution and Diversity, Goethe University, Max-von-Laue-Str. 13, 60438 Frankfurt am Main, Germany; 4 Senckenberg Naturhistorische Sammlungen Dresden, Museum für Tierkunde, Königsbrücker Landstraße 159, 01109 Dresden, Germany; 5 Department of Bioinformatics and Genetics, Swedish Museum of Natural History, Frescativägen 40, 114 18 Stockholm, Sweden; 6 Federal Scientific Center of the East Asia Terrestrial Biodiversity, Russian Academy of Sciences, Stoletiya Avenue 159, 690022 Vladivostok, Russia; 7 Institut für Organismische und Molekulare Evolutionsbiologie (iomE), Johannes Gutenberg-Universität Mainz, 55099 Mainz, Germany; 8 Department of Ornithology, Zoological Museum of Moscow State University, Bol’shaya Nikitskaya Street 2, 125009 Moscow, Russia; 9 Institute of Zoology, Chinese Academy of Sciences, CN-100101 Beijing, PR China; 10 Leibniz Institute for the Analysis of Biodiversity Change, Zoological Research Museum Alexander Koenig, Adenauerallee 127, 53113 Bonn, Germany

**Keywords:** Phylogenetics, Coevolution

## Abstract

Bird-mediated seed dispersal is crucial for the regeneration and viability of ecosystems, often resulting in complex mutualistic species networks. Yet, how this mutualism drives the evolution of seed dispersing birds is still poorly understood. In the present study we combine whole genome re-sequencing analyses and morphometric data to assess the evolutionary processes that shaped the diversification of the Eurasian nutcracker (*Nucifraga*), a seed disperser known for its mutualism with pines (*Pinus*). Our results show that the divergence and phylogeographic patterns of nutcrackers resemble those of other non-mutualistic passerine birds and suggest that their early diversification was shaped by similar biogeographic and climatic processes. The limited variation in foraging traits indicates that local adaptation to pines likely played a minor role. Our study shows that close mutualistic relationships between bird and plant species might not necessarily act as a primary driver of evolution and diversification in resource-specialized birds.

## Abstract (web summary)

Genomic and phylogeographic analyses indicate that resource-specialization did not play a major role in the diversification and speciation of seed dispersing nutcrackers

## Introduction

Seed dispersal by birds is an essential ecological process that contributes to the regeneration and viability of plant communities^1–3^. The resulting mutualistic relationship between seed-dispersing birds and their food plants potentially leads to resource specialization and can subsequently act as a driving force in speciation^2,4–7^. Nutcrackers (genus *Nucifraga*) exemplify a case of mutualism and are known for their preference for caching pine seeds. Seeds that are not retrieved have the potential to germinate, thus making nutcrackers a crucial vector in pine seed dispersal^8–10^. Although nutcrackers have been reported to collect and cache the seeds of spruce (*Picea*) and hazel (*Corylus*)^11,12^ as well, they primarily prefer the seeds of white pines (*Pinus*, subgenus *Strobus*)^13,14^. This pine subgenus is characterized by wingless, nutrient-rich nuts and relies on animals to disperse their seeds as the pine cones of the majority of these species do not open at maturity^13,15–17^. The mutualistic relationship between nutcrackers and white pines has been extensively studied between the Nearctic Clark’s nutcracker (*Nucifraga columbiana*) and North American pines^13,18,19^ and has similarly been described for Eurasian nutcrackers and European and Asian pines^10,20–25^. Furthermore, white pines are distributed across a large geographic range^26^ and have deep divergences^27,28^. Yet their role in the diversification of nutcrackers remains unexplored.

Nutcrackers fulfil an essential role in the maintenance and viability of pine ecosystems by actively dispersing their seeds. Yet, despite their ecological importance, little is known about the evolution of nutcrackers and the processes that shaped their diversification. The variation in beak morphology previously reported between subspecies indicates that nutcrackers adapted to different pine species throughout their range and supports the hypothesis that their evolution was driven by its mutualism with pines^8,21,29,30^. Subsequently, beak morphology has been a primary trait on which subspecies have been distinguished, such as the “slender-billed” (*N. c. macrorhynchos*) and “thick-billed nutcrackers” (*N. c. caryocatactes*)^29,30^. However, the taxonomic classification of the Eurasian nutcrackers has been plagued by inconsistency and until now has solely been based on phenotypic descriptions between (sub)species, acknowledging either one (*N. caryocatactes*^29,31^), two (*N. caryocatactes* and *N. multipunctata*^32–35^) or three (*N. caryocatactes*, *N. multipunctata*, *N. hemispila*^36^) Eurasian nutcracker species. In the current paper, we follow the most recent classification of Madge et al. and Gill et al.^34,35^ which implies two species (*N. caryocatactes* and *N. multipunctata*) with eight defined subspecies in *N. caryocatactes*. Additionally, Madge et al.^35^ recognize two distinct groups within *N. caryocatactes* (northern and southern group), without taxonomic rank.

Whether the diversification of nutcrackers was indeed driven by their link with pines remains unclear, and understanding their evolution requires a combined effort of distribution, morphometric and genomic data. So far, a genomic assessment of the nutcracker species complex remains absent and the few studies that analysed the diversification of nutcrackers were based on mitochondrial markers limited to populations in the Northern Palearctic region^37,38^. Hence, a comprehensive genome-wide sampling covering the complete nutcracker range is needed to gain a deeper understanding of the driving forces behind the diversification of Eurasian nutcrackers.

In the current study, we combined both whole-genome re-sequencing and morphometric data, covering the complete Eurasian nutcracker distribution range, to examine what processes shaped the evolution of the nutcracker species complex. We show that beak morphology only marginally contributed to the overall phenotypic variation between (sub)species and that the phylogeographic pattern of Eurasian nutcrackers resembles those of other Sino-Himalayan and Palearctic non-mutualistic passerine birds. We therefore conclude that the early diversification of nutcrackers was most likely driven by environmental and climatic forces, instead of a strong mutualism with pines.

## Results

### Genetic, phylogenomic and morphometric comparison between the Eurasian nutcracker species

**Fig. 1:**
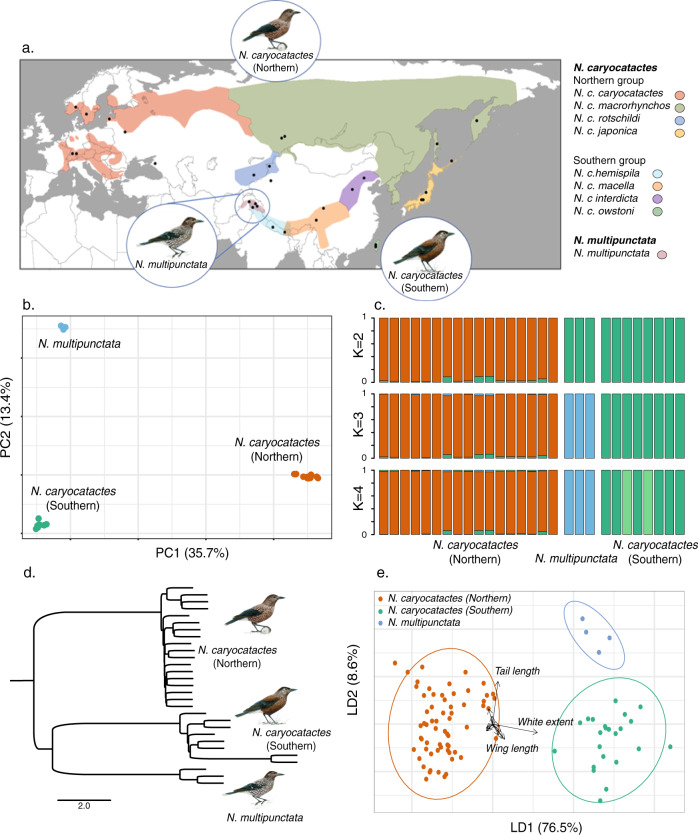
Genomic and morphometric analyses of the Eurasian nutcracker species complex. **a** Species range according to Madge et al.^35^ and Gill et al.^34^ with sampling locations (black dots) of *N. caryocatactes* (northern) (*N* = 17), *N. caryocatactes* (southern) (*N* = 8) and *N. multipunctata* (*N* = 3). Subspecies distributions are depicted in different colours according to the legend. **b** Principal component analysis based on 110,979 SNPs separated the data in three distinct clusters. **c** Population structuring based on admixture analysis for K ranging from 2 to 4. Cross-validation indicates K = 3 as the optimal number of clusters. **d** Species tree under the Multiple Species Coalescence model constructed from 192 nuclear genomic alignments of 1 Mbp. **e** Linear discriminant analysis of phenotypic variation among Eurasian nutcrackers based on an independent morphometric dataset of 90 museum specimens. Bird images by Jon Baldur Hlidberg, courtesy of the authors.

Principal component analysis (PCA) based on 110,979 linkage-pruned single nucleotide polymorphisms (SNPs) identified three distinct genetic clusters among the 31 re-sequenced Eurasian nutcracker samples (Fig. 1a, Fig. 1b and Supplementary Fig. 1). The first principal component explained 35.7% of the variation and separated the northern *N. caryocatactes* group from both *N. multipunctata* and the southern *N. caryocatactes* group, whereas the second principal component explained 13.4% of the variation and separated *N. multipunctata* from the other two groups. Admixture analyses based on the same SNP dataset with ancestry components (K) ranging from 2 to 4 supported this pattern and identified K = 3 as the optimal number of clusters (Fig. 1c and Supplementary Fig. 2).

Maximum likelihood (ML) phylogenetic inference was performed based on whole mitochondrial (mt) genome alignments, revealing three distinct evolutionary lineages corresponding to a northern *N. carycocatactes* clade, southern *N. caryocatactes* clade and *N. multipunctata* clade. However, the internal node received a low support value and therefore reflects a poorly resolved tree (Supplementary Fig. 3). Maximum likelihood phylogenetic inference and multispecies coalescent (MSC) species tree reconstruction of the nuclear genome based on 192 non-overlapping 1 Million base pairs (Mbp) alignments similarly revealed three distinct genetic lineages and supported a topology in which *N. caryocatactes* (northern) is a sister group to *N. caryocatactes* (southern) and *N. multipunctata* (Fig. 1d, Supplementary Figs. 4 and 5). However, with a normalized quartet score of 71.65%, the MSC species tree indicates conflicting phylogenetic signals within the nuclear genome.

**Fig. 2:**
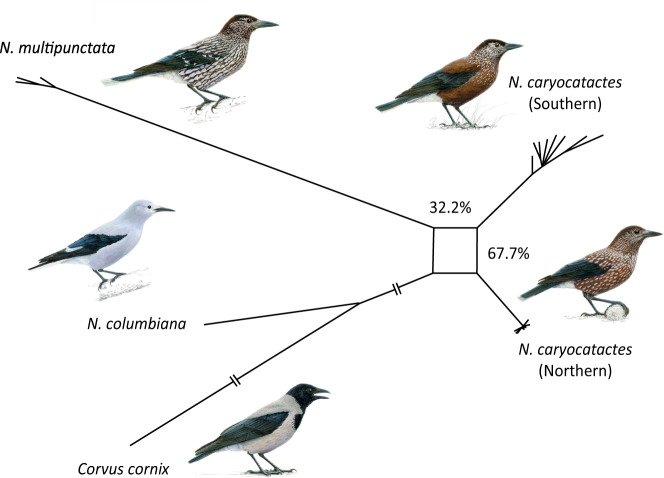
Evolutionary network of the four nutcracker species and their sisterspecies. Phylogenetic network of the Eurasian nutcracker species complex based on 192 alignments of 1 Mbp of the nuclear genome. The numbers at the branches indicate the percentage of genomic alignments supporting the split. Some branch lengths are shortened for visualization purposes. Bird images by Jon Baldur Hlidberg, courtesy of the authors.

To assess potential reticulation as a cause of the discordance between the mitochondrial and nuclear data and low node support value, we subsequently constructed a phylogenetic network based on the 192 alignments of 1 Mbp, which consistently revealed genomic conflict for the deepest divergences between nutcrackers for thresholds ranging between 0.01 and 0.30 thus depicting reticulate evolution for alternative branches that were supported by up to 30% of the 192 alignments (Fig. 2). One third (32.2%) of the alignments supported the split of *N. multipunctata* from *N. caryocatactes* (northern) and *N. caryocatactes* (southern), whereas 67.7% of the 1 Mbp alignments supported the split of *N. caryocatactes* (northern) from *N. caryocatactes* (southern) and *N. multipunctata*.

Divergence time estimates of the Eurasian nutcracker species complex were based on the mtDNA alignment of 59 individuals covering 31 taxa (Supplementary Fig. 6 and Supplementary Table 1). The nutcrackers and crows diverged in the late Miocene at ~8.00 (5.74−10.20) million years ago (Ma), whereas the Nearctic Clark’s nutcracker (*N. columbiana*) and Eurasian nutcrackers diverged during the late Miocene/early Pliocene at around ~4.72 (3.2–6.23) Ma. Within the Eurasian nutcracker species complex, the deepest split occurred during the late Pliocene/early Pleistocene at ~2.32 (1.59–3.11) Ma and the youngest split at ~1.87 (1.17–2.60) Ma (*N. caryocatactes* northern and *N. multipunctata*). The within-species divergence occurred only recently at ~340–50 kilo years ago (ka) (Supplementary Fig. 7).

Phenotypic variation was examined based on an independent morphometric dataset of 90 museum specimens. First, a Principal Coordinate Analysis (PCoA) was performed based on 12 phenotypic traits to assess the morphometric variation within the data without any prior group assumptions (Supplementary Figs. 8 and 9). Results indicate two clusters, separating the northern *N. caryocatactes* group from the southern *N. caryocatactes* group and *N. multipunctata*. This variation was primarily driven by wing length and white extent on the tail feathers. In contrast, linear discriminant analysis (LDA) based on a priori defined groups revealed the presence of three distinct clusters (Fig. 1e, Supplementary Fig. 10). The first linear discriminant accounted for 76.52% of the phenotypic variation and was similar to the PCoA primarily characterized by variation in white extent on the tail feathers, separating the northern *N. caryocatactes* group from the southern *N. caryocatactes* group and *N. multipunctata* (Supplementary Table 2). However, the second linear discriminant explained 8.63% of the variation, separating *N. multipunctata* from both the northern and southern *N. caryocatactes* groups, and was characterized by variation in tail length. Comparison of beak traits revealed only a weak yet significant difference in bill morphology (Welch’s *t*-test *p* ≤ 0.05), with shorter and higher bills in the southern *N. caryocatactes* nutcrackers compared to the northern *N. caryocatactes* nutcrackers (Supplementary Fig. 11).

### Genetic, phylogenetic and morphometric comparison between Eurasian nutcracker subspecies

**Fig. 3:**
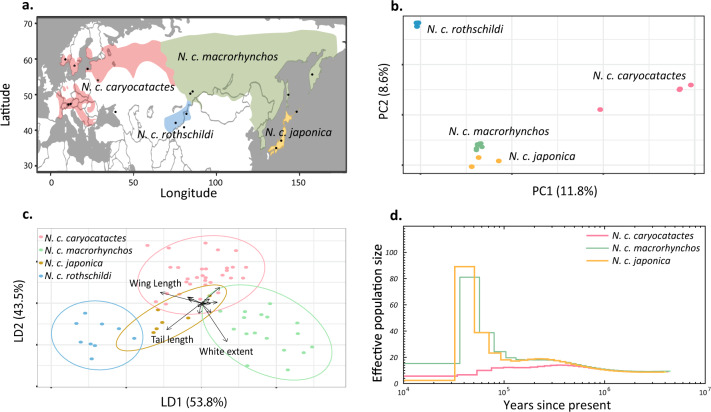
Genetic and morphometric differentiation within the northern *N. caryocatactes* group. **a** Distribution of the four subspecies within the northern *N. caryocatactes* group. Two specimens sampled in the range of *N. c. caryocatactes* were taxonomically and genetically identified as *N. c. macrorhynchos*. **b** PCA based on 142,799 SNPs separated the northern *N. caryocatactes* nutcrackers in three clusters. The specimen located in the middle of *N. c. caryocatactes* and *N. c. macrorhynchos*/*N. c. japonica* reflects a potential F1-hybrid. **c** Linear discriminant analyses based on morphometric measurements of 63 northern *N. caryocatactes* museum specimens. **d** Reconstruction of historical effective population size through time based on PSMC. *N. c. rothschildi* was excluded due to limited genome coverage.

**Fig. 4:**
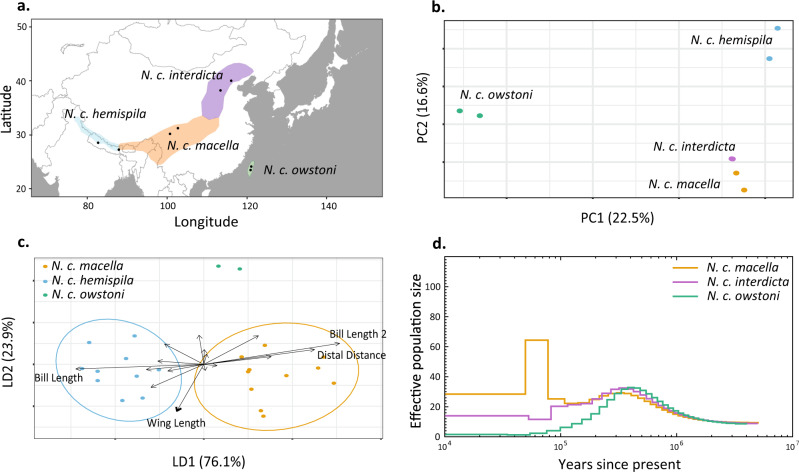
Genetic and morphometric differentiation within the southern *N. caryocatactes* group. **a** Distribution of the four subspecies within the southern *N. caryocatactes* group. **b** Within-species genetic variation based on a PCA of 101,611 SNPs. **c** Linear discriminant analyses based on morphometric measurements of 23 southern *N. caryocatactes* museum specimens. *N. c. interdicta* had to be excluded due to lack of trait data. **d** Reconstruction of historical effective population size through time based on PSMC. *N. c. hemispila* was excluded from the analyses due to insufficient genome coverage.

We performed both PCA and admixture analyses for the northern and southern *N. caryocatactes* groups separately to examine genetic differentiation at the subspecies level (Figs. 3 and 4). PCA based on 142,799 SNPs identified three clusters within the northern *N. caryocatactes* group (Fig. 3b). The first principal component explained 11.8% of the variation and separated the European subspecies (*N. c. caryocatactes*) from the other subspecies. The second principal component explained 8.6% of the variation and separated the Central Asian subspecies (*N. c. rothschildi*). No clear genetic differentiation was found between the Siberian (*N. c. macrorhynchos*) and Japanese (*N. c. japonica*) subspecies. The admixture analyses reflected a similar pattern as the PCA for K ranging from 2 to 4. However, cross-validation tests suggest K = 2 as the most likely number of genetic clusters, only assigning *N. c. caryocatactes* to its own genetic group (Supplementary Figs. 2 and 12). Both the PCA and admixture analyses identified one individual as a likely F1-hybrid between *N. c. caryocatactes* and *N. c. macrorhynchos*.

PCoA of morphometric traits revealed no clear variation between groups (Supplementary Figs. 8 and 9) and a LDA based on a priori defined groups revealed only marginal phenotypic differentiation among subspecies, as indicated by the overlap in clusters (Fig. 3c). The main phenotypic traits that contributed to the linear discriminants consisted of wing length, white extent on the tail feathers and variation in tail length (Supplementary Table 3). Trait-specific analyses of beak morphology revealed significant variation between subspecies, with *N. c. caryocatactes* having wider bills and *N. c. japonica* shorter bills compared to the other northern *N. caryocatactes* subspecies (Supplementary Fig. 13).

Reconstruction of the demographic history using pairwise sequentially Markovian coalescent (PSMC) suggests that the Siberian (*N. c. macrorhynchos*) and Japanese (*N. c. japonica*) nutcrackers underwent a sharp increase in effective population size (N_e_) around 50–100 ka ago, followed by a rapid decrease around 30–40 ka ago (Fig. 3d, Supplementary Figure 14). In contrast, the European population (*N. c. caryocatactes*) had a low and continuously decreasing N_e_ throughout the past 400,000 years. No demographic estimates were included for *N. c. rothschildi* due to insufficient genome coverage.

PCA based on 101,611 SNPs identified three clusters within the southern *N. caryocatactes* group. The first principal component explained 22.5% of the variation and separated the Taiwanese subspecies (*N. c. owstoni*) from the other subspecies (Fig. 4b). The second principle component explained 16.6% of the variation and separated the subspecies of North-West/Central Himalayas (*N. c. hemispila*) from the other subspecies. The two subspecies in South and North China showed strong genetic similarity (*N. c. macella* and *N. c. interdicta*). Admixture analyses with K ranging from 2 to 4 identified distinct genetic groups for each of the subspecies at K = 4 (Supplementary Fig. 12), though cross-validation tests suggest K = 2 as the most likely number of clusters (Supplementary Fig. 2).

Similar to the northern group, the PCoA of the morphometric traits revealed no clear variation between the southern groups (Supplementary Figs. 8 and 9). However, LDA identified *N. c. macella* and *N. c. hemispila* as distinct subspecies (Fig. 4c). The first linear discriminant explained 76.1% of the variance and was mainly driven by differences in bill length and the distal distance of the wings (distance between the tip of outermost primary and tip of the wing), whereas the second linear discriminant explained 23.9% of the variance and was primarily driven by variation in wing length, separating *N. c. owstoni* from the other two subspecies. However, none of the traits differed significantly between subspecies (Supplementary Table 4). Measurements for *N. c. interdicta* were incomplete and had to be excluded from this analysis. Separate analyses of beak morphology revealed no significant variation in beak traits between the southern *N. caryocatactes* subspecies (Supplementary Fig. 13).

Analyses of the historical effective population size revealed a continuous decline in N_e_ of the Taiwanese subspecies (*N. c. owstoni*) from 400 ka ago until present (Fig. 4d and Supplementary Fig. 14). Both the southern and northern Chinese subspecies (*N. c. macella* and *N. c. interdicta*) underwent a decrease in N_e_ around 400–100 ka ago. However, *N. c. macella* underwent a rapid increase and decrease in N_e_ around 100–60 ka ago, whereas *N. c. interdicta* continued to decrease in the same period. Both populations seem to have stabilized afterwards. No demographic estimates were included for *N. c. hemispila* due to insufficient coverage in both samples.

## Discussion

In the current study, we used a whole-genome sequencing approach in combination with morphometric analyses to assess the evolutionary and phylogeographic history of the Eurasian nutcracker, a food caching corvid known for its mutualism with pines. The results presented here provide clear evidence that the Eurasian nutcracker species complex is characterized by three evolutionary lineages, corresponding to a widespread northern Palearctic clade (*N. caryocatactes* (northern)), a widespread Sino-Himalayan clade (*N. caryocatactes* (southern)) and a narrow-range endemic Western Himalayan clade (*N. multipunctata*). Our data supports three species-level taxa of Eurasian nutcrackers in accordance with previous classifications recognizing the southern group as its own species *N. hemispila*^36^, warranting a taxonomic revision. Divergence time estimates placed the deepest divergences of the Eurasian nutcrackers at 2.32 Ma, followed by a second split around 1.87 Ma (Supplementary Figs. 6 and 7). However, our analyses could not fully resolve the phylogenetic relationship between the three lineages, most likely due to the short time span between the two speciation events which resulted in high levels of incomplete lineage sorting^39,40^. Past introgressive gene flow may complicate the phylogenetic reconstruction of nutcrackers, but given the unresolved topology, the hypothesis is with current methods not possible to examine. The evolutionary history of the Eurasian nutcracker species complex therefore seems to be characterized by a rapid speciation event that occurred at the beginning of the Pleistocene. Rather than a bifurcating phylogeny, the phylogenetic history of the Eurasian nutcracker species complex is instead better depicted as a reticulate network (Fig. 2), a pattern that seems to become common for many avian species with the increasing number of whole-genome studies^40–43^.

The spatial diversification pattern of nutcrackers is rather different from those found in other corvids^44–46^. Many Palearctic corvid species do not have any close relatives in the (Sino-)Himalayas^37,47^, whereas several Sino-Himalayan corvid species represent an Indomalayan diversification without any closer affiliation to the Palearctic^48^. Instead, the divergence times and phylogeographic distribution of nutcrackers seem to closely match those of other montane passerines of the temperate forest belt in the Sino-Himalayas, particularly for those clades encompassing endemics of the Western Himalayan subalpine conifer forests. Of the randomly distributed 192 non-overlapping 1 Mbp alignments of the nuclear genome, 67.7% suggested a phylogenetic relationship characterized by close relatedness between the Western Himalayan and Sino-Himalayan nutcrackers which formed a sister group to the northern Palearctic nutcrackers (Figs. 1d, 2, Supplementary Figs. 15 and 16), a phylogeographic pattern which is common among Sino-Himalayan passerine birds^49–53^ (Supplementary Fig. 17). The alternative phylogeographic pattern in which the northern Palearctic and Sino-Himalayan nutcrackers form a sister group to the Western-Himalayan nutcrackers was less frequently supported by the genomic analyses (Fig. 2, Supplementary Figs. 15 and 16). This phylogeographic pattern has only previously been found in leaf warblers (e.g. *Phylloscopus subviridis*)^54^ and therefore seems to be less common among Sino-Himalayan birds. Overall, the similarity in phylogeographic patterns and divergence times between the nutcrackers and Sino-Himalayan passerines, suggests that similar environmental and climatic forces have shaped the diversification of these species. Future studies utilizing ecological niche modelling would be important to better understand these phylogeographic patterns on a broader scale.

The northern Palearctic region is characterized by an East-West (E-W) phylogeographic pattern for passerine birds, caused by strong Pleistocene climatic changes and has been observed in several corvid species^37,45,46,52,55–59^. Similarly, our genome-wide analyses showed a clear E-W disjunction between the European nutcrackers and the other northern Palearctic nutcrackers, in contrast to previous studies which showed no clear E-W divergence based on the mitochondrial control region^37,38^. Additionally, one hybrid individual identified in our dataset originated from Sweden and suggests ongoing gene flow between the European and Siberian nutcrackers, likely linked to frequent mass irruptive migrations of Siberian nutcrackers into Western Europe during times of food scarcity^11,12,31,60^. In Sweden, local colonies of Siberian nutcrackers (*N. c. macrorhynchos*) became established after the invasion event of 1977^61^ which would explain the occurrence of admixed individuals in that area. Interestingly, both the Japanese (*N. c. japonica*) and Siberian nutcrackers (*N. c. macrorhynchos*) showed strong genetic similarity and overlap in their historical effective population size, despite their large distribution range, suggesting either a panmictic ancestral population in shared refugia^37,62,63^ or extensive gene flow and migration during the late Pleistocene via the existence of land bridges between Japan and continental Eurasia^64^.

Our analyses could similarly identify another clear E-W phylogeographic disjunction in the Sino-Himalayan nutcrackers, separating the North-West/Central Himalayan nutcrackers (*N. c. hemispila*) from those of South-West China, originating around 300–100 ka. The divergence seems to have co-occurred with cyclic contractions and expansions of conifer forests during the late Pleistocene^65^ and reflects a phylogeographic pattern found in other montane passerine birds of the Sino-Himalayas^51,66^. Furthermore, the Taiwanese subspecies (*N. c. owstoni*) was genetically distinct compared to the other Sino-Himalayan nutcrackers and together with the continuously decreasing effective population size over the last 300–400 ka, reflects a loss of genetic diversity typically found in island populations as a result of drift, inbreeding and genetic isolation^67^.

Despite previous reports of phenotypic variation in beak morphology between nutcracker subspecies, it is still unclear whether the mutualism between nutcrackers and pines resulted in co-evolutionary processes in which local adaptation to pine cone morphology was the driving factor of beak variation. For example, crossbills (*Loxia*) illustrate a typical co-evolving arms-race with lodgepole pines (*Pinus contorta latifolia*) where divergent selection on beak morphology contributes to reproductive isolation of populations resulting in different crossbill species^6,68,69^. Moreover, only limited genome-wide differentiation was found between crossbill ecotypes, present only at a small number of loci that arose over as little as 6000 years^6,7^. If nutcrackers represent a similar case of co-evolution, we would expect that phenotypic differentiation between subspecies was primarily driven by variation in beak morphology. Our analyses show that variation in beak morphology between nutcracker species only contributed marginally to the phenotypic variation between species. Phenotypic diversification of the three main Eurasian nutcracker lineages was primarily driven by variation in flight-related traits (e.g. tail length) as well as in the white extent on the tail feathers, thus suggesting selection pressures unrelated to food resources as a driver for phenotypic diversification.

At the subspecies level, the phenotypic variation of northern Palearctic nutcrackers was similarly driven by variation in flight-related traits and white extent on the tail feathers. No significant morphological variation was found between the Sino-Himalayan subspecies, though the low number of samples and lack of *N. c. interdicta* in the dataset requires more extensive sampling to fully understand the phenotypic variation within the Sino-Himalayan nutcrackers. Yet, these initial results seem to support the idea that variation in beak morphology only marginally contributed to the phenotypic differentiation between species and subspecies and that flight-related traits are driving diversification. An extended in-depth analysis focussing only on beak traits revealed significant variation between several northern Palearctic subspecies. For example, the “Thick-billed” nutcracker (*N. c. caryocatactes*) had significantly wider bills compared to the other subspecies, which has previously been linked to their preference for both hazelnuts and pine seeds^29,30,70^. Interestingly, the Japanese nutcrackers had a significantly shorter beak compared to the other northern Palearctic subspecies, despite the lack of genome-wide differentiation with Siberian nutcrackers (Fig. 3b and Supplementary Fig. 13). As was shown in the case of *Loxia* crossbills, as well as in Darwin finches^71,72^, diversification of ecotypes can occur in short timescales without genome-wide diversification. It is therefore possible that the Siberian and Japanese nutcrackers only recently became adapted to different food sources. Indeed, the Japanese nutcrackers have been linked with Japanese stone pine (*Pinus pumila*)^23,24^ while the Siberian nutcracker feeds preferentially on Siberian pine (*Pinus sibirica*)^73^, which typically has longer seeds compared to Japanese stone pines (10–14 mm vs 7–10 mm, respectively)^17,74^. Additionally, some nutcracker subspecies occur in regions not occupied by pine species and have most likely adapted to different food sources, such as Asian spruce (*Picea schrenkiana*) in the case of *N. c. rothschildi* (Supplementary Fig. 1 d). Combined with the known preference of *N. c. caryocatactes* for both pine and hazel seeds, nutcrackers therefore seem to be characterized by a more opportunistic feeding strategy and support the hypothesis that local adaptation to pines played only a minor role in the diversification of these birds. However, subsequent studies are needed to directly link the variation in nutcracker beaks to the pine cone and pine seed morphology. Furthermore, more extensive genomic sampling is needed in order to link beak traits to specific genomic regions^71,72^ to get a deeper understanding of the underlying genetic basis of beak variation in nutcrackers.

Although nutcrackers are characterized by a strong mutualism with pines, our results show that resource specialization and subsequent local adaptation resulted in only minor phenotypic differentiation between nutcracker lineages. The strongest overall differences between species lie in flight-related traits rather than beak characters. Furthermore, the divergence times and phylogeographic patterns of nutcrackers were highly congruent with those of other non-mutualistic passerine birds, suggesting that similar evolutionary and climatic forces shaped the evolution of these species. Divergence time estimates could link these to climatic fluctuations during the Pleistocene period. Our study therefore suggests that a mutualistic relationship between plants and animals does not necessarily need to coincide with strong selective pressures on speciation and can instead be characterized by a high degree of evolutionary flexibility where other processes such as climatic and environmental factors are more determinant in their diversification.

## Materials and methods

### De novo assembly

As a basis for the genomic analyses, a reference nutcracker genome was assembled using 10X Genomics linked read technology. A blood sample from a northern *N. caryocatactes* specimen (Voucher ID: NHMO-BI-40098/1-B Natural History Museum of Oslo) was sent to Beijing Genomic Institute (BGI) Hong Kong for DNA extraction and library preparation. Sequencing was subsequently conducted on a single Illumina HiSeq X Ten lane (2 × 151 bp), yielding a total of 88.9 Gbp of raw sequence data.

Quality control of raw read data was performed with FastQC v0.11.7^75^ and subsequent *de novo* assembly of the genome was performed with the 10X genomics Supernova v.1.0.0 assembly pipeline^76^. The quality of the assembly was assessed with QUAST v. 6.4.3^77^ and gene completeness was assessed with BUSCO v.3.0.2^78^ using the Aves gene set (available at busco.ezlab.org). Synteny differences between the *Nucifraga* assembly and the collared flycatcher (*Ficedula albicollis*) assembly (acc. No. GCA_000738735.2) were analysed with JupiterPlot^79^ (Supplementary Fig. 18). For downstream analyses, scaffolds smaller than 100 Kbp were filtered out, with the exception of those that contained BUSCO genes. The final assembly had a total length of 1.14 Gbp, with a contig N50 of 217.12 Kbp and scaffold N50 of 12.61 Mbp (Supplementary Table 5). BUSCO assessment identified 93.4% complete, 4.0% fragmented and 2.6% of missing genes of the Aves set (*n* = 4915).

RepeatModeler v.1.0.11^80^ was used to identify de novo repeats in the nutcracker genome. The resulting de novo repeat database was merged with the custom annotated repeat library used for studying crows^81^. The custom repeat library was subsequently used to mask repetitive sequences in the nutcracker genome with RepeatMasker v.4.1.0^82^. Repetitive regions comprised 9.23% of the genome (Supplementary Table 6).

### Re-sequencing, mapping and genotype calling

In the current paper, we have assumed the classification of Madge et al.^35^ and Gill et al.^34^ that acknowledge two Eurasian nutcracker species (*N. caryocatactes* and *N. multipunctata*) with eight defined subspecies in *N. caryocatactes*. Additionally, Madge et al.^35^ recognize two distinct groups within *N. caryocatactes* (northern and southern group), though they do not officially acknowledge them as independent species. We therefore sampled a total of 31 individuals covering each species and including at least a minimum of two individuals per subspecies (Fig. 1a). Additionally, one individual from the closely related Clark’s nutcracker (*Nucifraga columbiana*) was sequenced to use as an outgroup for the phylogenomic analyses. Samples consisted of either blood, tissue or toe pads, originating from varying natural history collections (Supplementary Table 7). DNA from tissue and blood samples was extracted with the Macherey-Nagel NucleoSpin® Tissue kit according to the manufacturer’s protocol. DNA from museum samples was extracted from 3 × 3 mm toe pads^83^. Sequencing libraries were prepared with 300 bp insert-sizes and sequenced with the Illumina Hiseq X technology at BGI (HongKong) or at the National Genomics Institute in Stockholm (SciLife). Additionally, we retrieved publicly available short-read sequencing data for the closely related hooded crow (*Corvus cornix*) (Acc. SRR1266946 -SRR1266949) as an additional outgroup.

Reads were processed using a custom-designed, clean-up workflow (https://github.com/mozesblom). Cleaning steps included quality control with FastQC v0.11.7, deduplication with Super-Deduper v2.0 (https://github.com/dstreett/Super-Deduper), trimming with Trimmomatic v.0.38 set at SLIDINGWINDOW:4:20 and MINLEN:30^84^, merging of overlapping reads with PEAR v0.9.6^85^ for reads that overlapped for ≥20 base pairs and removal of low complexity reads with a cut-of threshold of ≥0.5. The resulting reads were mapped to the de novo nutcracker genome with the default settings of BWA mem v.0.7.17^86^ (Supplementary Table 8), and duplicates were marked with Picard v.2.18.21 (http://broadinstitute.github.io/picard/). Subsequently, we used freebayes v1.3.1^87^ to perform variant calling with the following settings:-report-monomorphic –min-mapping-quality 20 -C4 and -F 0.3. For the whole-genome alignments, indels were removed and consensus sequences were created from the VCF files using custom perl scripts (https://github.com/mobilegenome/phylogenomics/), masking out ambiguous sites with N’s and removing all positions with missing data.

### Phylogenetic analyses

We reconstructed the mitochondrial genomes for all individuals by mapping the cleaned reads to the mitochondrial genome of the Clark’s nutcracker (*Nucifraga columbiana*) (KF509923) using BWA mem, followed by variant calling with freebayes^87^, using the same settings as described above for the nuclear genome. Bcftools’ consensus function was then used to call consensus sequences. Mitochondrial genomes were aligned with MAFFT v7.407 (settings L-INS-i) and we included previously published mitochondrial genomes of the Magpie (*Pica pica*—HQ915867) and hooded crow (*Corvus cornix*—CM002877). Subsequent Maximum Likelihood tree inference was performed with IQ-TREE v.1.6.11 using the optimal substitution model identified by ModelFinder^88^ implemented in IQ-TREE^89^.

Phylogenomic inference of the nuclear genome proceeded using a method similar to a non-overlapping sliding window approach^90^. First, consensus sequences were generated for each individual, followed by the removal of heterozygous sites and N’s for all individuals using bedtools v2.28.0. Sequences were subsequently aligned per scaffold with custom python scripts (https://github.com/mobilegenome/phylogenomics/). To identify the minimum nucleotide length of the sliding windows that carry sufficient phylogenetic information to reject alternative topologies, increasingly longer windows were analysed by using an approximately unbiased (AU) test^91^ as implemented in IQ-TREE v.1.6.11 (Supplementary Figs. 15 and 16). The AU test was performed with 10,000 replicates for window lengths of 50 Kbp to 2 Mbp in steps of 50 Kbp, with 100 randomly sampled alignments per window length. Of the three potential nutcracker topologies that were tested with the AU test, one could be significantly rejected when windows had a length of ≥1 Mbp. Increasing the window length further could not significantly accept or reject the other two potential topologies (Supplementary Fig. 16). Subsequently, the scaffold alignments were split into 1 Mbp non-overlapping windows and only those windows that contained sufficient phylogenetic content (>2000 informative sites) were retained, resulting in a total of 192 1 Mbp alignments (192 Mbp, representing 19.6% of the repeat-filtered genome).

Maximum Likelihood trees were inferred for each 1 Mbp alignment in IQ-TREE using 1,000 ultrafast bootstrap replicates and the implemented automatic ultrafast model selection. From the resulting trees, we constructed a multispecies coalescent species tree using ASTRAL-III v5.6.1^92^. The trees were rooted with Clark’s nutcracker and hooded crow as outgroups. Lastly, a consensus network was generated based on the 192 inferred ML trees. The “Consensus Network” method was used as implemented in SplitsTree v.4.15.1^93^ with a minimum proportion of trees supporting the splits ranging between 0.05 and 0.4.

### Allele-based analyses

VCF files were filtered for biallelic positions and SNPs were removed when coverage was less than 8× or more then 2.5 times the mean coverage for that particular individual. To account for the bias resulting from mapping to an in-group reference genome and the biases introduced by combining both museum and fresh samples in our study, we applied a strict filtering approach in which at maximum one individual was allowed to have missing data at each site. The final number of SNPs within the total dataset was reduced from 11,602,883 to 837,481 SNPs after filtering. Two allele frequency-based analyses were implemented to examine the genetic variation within the data. First, a PCA was performed between and within species with Plink version 1.9^94^. To account for linkage disequilibrium, sites within a 50-SNP stepping window with a correlation coefficient higher than 0.1 were omitted. Secondly, ADMIXTURE v1.3.0^95^ was used to estimate the population structure within the data. The VCF-file was converted to plink’s PED format with VCFtools and admixture analyses were run for K = 2 to K = 4. ADMIXTURE’s cross-validation procedure was used to determine the optimal number of clusters.

### Divergence time estimation

Divergence times were estimated using BEAST2 (version 2.7.0)^96^. The bmodeltest^97^ with a transition-transversion split was used to find a fitting substitution model. The mitochondrial protein-coding genes were extracted and aligned using MAFFT v7.407 (settings L-INS-i)^98^ and manually inspected. The ND6 gene was excluded from the dataset due to being encoded on the opposite strand. The final alignment of 10,851 nucleotides long included 59 individuals, covering 31 species (Supplementary Table 9). Five fossil-based calibration points were selected from the literature^99,100^ and used to estimate the divergence times (Supplementary Table 1). Zero offset values were based on the minimum fossil ages and the log mean and standard deviation was adjusted to achieve the optimal match of the normal prior distribution for the fossil age interval^101^. Additionally, we applied a normal prior to the root age to avoid the occurrence of implausibly old root ages. All calibrated nodes were set to be monophyletic. Four independent Markov chain Monte Carlo (MCMC) were run for 100,000,000 generations sampling every 10,000. Trace files were examined with Tracer v1.7.1^102^ to ensure chain convergence and appropriate effective sample sizes (ESS > 200). The tree log files from each iteration were combined with LogCombiner with a 50% burn-in and maximum clade credibility (MCC) tree was summarized in TreeAnnotator with a 20% burn-in. Both LogCombiner and TreeAnnotator are part of the BEAST2 package.

### Population size history

The demographic history was assessed with PSMC v.0.6.5^103^. Consensus sequences in FASTQ format were generated for each individual that had a coverage of ≥17X^104^ using the ‘mpileup’ command in BCFtools v.1.9 (https://samtools.github.io/bcftools/) and the included script ‘vcfutils.pl’. Sites with a read depth below 8 or above twice the sample’s median depth and a minimum mapping quality below 20 were removed. PSMC was run for 25 iterations with the upper limit of the TMRCA set to -t 5, the initial h/q value to -r 1, and 34 atomic time intervals (4 + 30*2 + 4 + 6 + 10), as is recommended for avian genomes^104^. We performed 100 bootstrap replicates by randomly sampling with replacement 1 Mbp blocks from the consensus sequence. Results were scaled using a mutation rate (m) of 3.18 × 10^−9^ substitutions per generation^81^ and a generation time of 7 years^105^ (Supplementary Fig. 14).

### Morphometric analyses

To assess the phenotypic differentiation among nutcrackers, we measured 12 morphological traits on nutcracker specimens from museum collections. We used morphological traits associated with nutcracker foraging and diet, flight ability, manoeuvrability, bipedal locomotion and plumage colour (detailed description of all measures in Supplementary Table 10). Traits were measured on a total number of 118 specimens from the Zoological Research Museum Alexander Koenig, Bonn, Germany, the Natural History Museum, Berlin, Germany, Senckenberg Natural History Collections, Dresden, Germany and the Senckenberg Forschungsinstitut und Naturmuseum, Frankfurt, Germany. First, phenotypic variation was assessed without a priori defined groups by implementing a PCoA as part of the ape package (v. 5.5)^106^ in R v. 3.5.2^107^. Subsequently, a LDA was performed to assess the morphometric variation between groups defined by our genomic analysis. Specimens with missing data were excluded from the analyses, resulting in a total sample size of *N* = 90. Similarly, a LDA was performed to assess morphometric variation at the subspecies level for both the northern *N. caryocatactes* (*N* = 63) and southern *N. caryocatactes* (*N* = 23) nutcrackers. LDA were performed with the ggord package^108^ in R v. 3.5.2^107^. Traits that contributed primarily to the linear discriminants were further examined. First, a Shapiro-Wilk test was performed to test whether the traits adhered to a normal distribution. Subsequently, a Welch’s *t*-test with Bonferroni correction was performed to compare means between groups. A similar approach was implemented to examine the variation in beak traits between groups.

Vegetation maps of the main food resources linked to nutcrackers^26^ were plotted with R^108^ (Supplementary Fig. 1 and Supplementary Table 11). In total the distribution of 26 plant species that have been linked to, or occur in the same geographical region as the nutcrackers were selected and plotted. This included two species of spruce (*Picea*), eight species of hazel (*Corylus*) as well as 16 species of pine (*Pinus*) that occur in the Palearctic region. All 11 species of white pines (*Pinus* subgenus *Strobus* Section *Quinquefoliae* subsection *Strobus*) occurring in the Palearctic region were selected.

### Statistics and reproducibility

Information about the statistical analyses is given in the methods section, results section and the supplementary file.

### Reporting summary

Further information on research design is available in the Nature Research Reporting Summary linked to this article.

## Supplementary information

Supplementary Information

Reporting Summary

## Data Availability

The data produced in the current study are available at the NCBI Sequence Read Archive under BioProject PRJNA682958 accession numbers SAMN17014695–SAMN17014724. The raw 10X genomics sequencing data used for the nutcracker reference assembly have been deposited at NCBI under accession number SAMN17050349. All supporting data are uploaded to Dryad, 10.5061/dryad.stqjq2c57^109^.

## Code availability

All software versions and scripts needed to reproduce the results are specified in the Methods.
