# Supplementary material for: Speciation and population divergence in a mutualistic seed dispersing bird
Source: Commun Biol. 2022 May 9;5:429. doi: 10.1038/s42003-022-03364-2 (PMC9085801; doi:10.1038/s42003-022-03364-2)
Supplement: Supplementary file 1 — Supplementary Information [file 42003_2022_3364_MOESM1_ESM.pdf]

Supplementary information

Speciation and population divergence in a mutualistic seed dispersing bird

Supplementary Figures

Supplementary Figure 1. Vegetation and distribution maps of plants associated with Nutcrackers

Supplementary Figure 2. Cross-validation error of admixture models

Supplementary Figure 3. Phylogenetic analyses based on mitochondrial genomes.

Supplementary Figure 4. Multispecies coalescence tree based on the nuclear genome.

Supplementary Figure 5. Maximum likelihood tree based on the nuclear genome.

Supplementary Figure 6. Divergence time estimation based on mitochondrial alignments.

Supplementary Figure 7. Divergence time estimates of the Eurasian nutcracker species complex.

Supplementary Figure 8. Principal coordinate analysis of morphometric traits

Supplementary Figure 9. Within-species principal coordinate analysis of morphometric traits

Supplementary Figure 10. Discrepancy between morphology and genetics in one specimen**.**

Supplementary Figure 11. Phenotypic variation in beak morphology between nutcracker species.

Supplementary Figure 12. Within-species genetic variation based on admixture analyses.

Supplementary Figure 13. Phenotypic variation in beak morphology between subspecies.

Supplementary Figure 14. Reconstruction of demographic history (PSMC).

Supplementary Figure 15. Possible topologies for the nutcracker species complex.

Supplementary Figure 16. Approximate unbiased (AU) test for phylogenetic inference.

Supplementary figure 17. Similarity in phylogeographic and divergence patterns between Sino-Himalayan passerines.

Supplementary Figure 18. Synteny between the assembled nutcracker genome and chromosome level flycatcher genome

Supplementary Tables

Supplementary Table 1. Calibration points and tMRCA prior settings used for molecular dating.

Supplementary Table 2. Measurements and comparisons of morphometric traits for the three main *Nucifraga* groups.

Supplementary Table 3. Measurements and comparisons morphometric traits within the northern *N. caryocatactes* group.

Supplementary Table 4. Measurements and comparisons morphometric traits within the southern *N. caryocatactes* group.

Supplementary Table 5. *Nucifraga* genome assembly summary statistics

Supplementary Table 6. Repeat content of the de novo assembled *Nucifraga* genome*.*

Supplementary Table 7. Sample collection information.

Supplementary Table 8. Short read mapping statistics

Supplementary Table 9. Additional taxa included in the divergence times estimation.

Supplementary Table 10. Phenotypic traits measured for the morphometric analyses.

Supplementary Table 11. Plant and bird associations and distributions

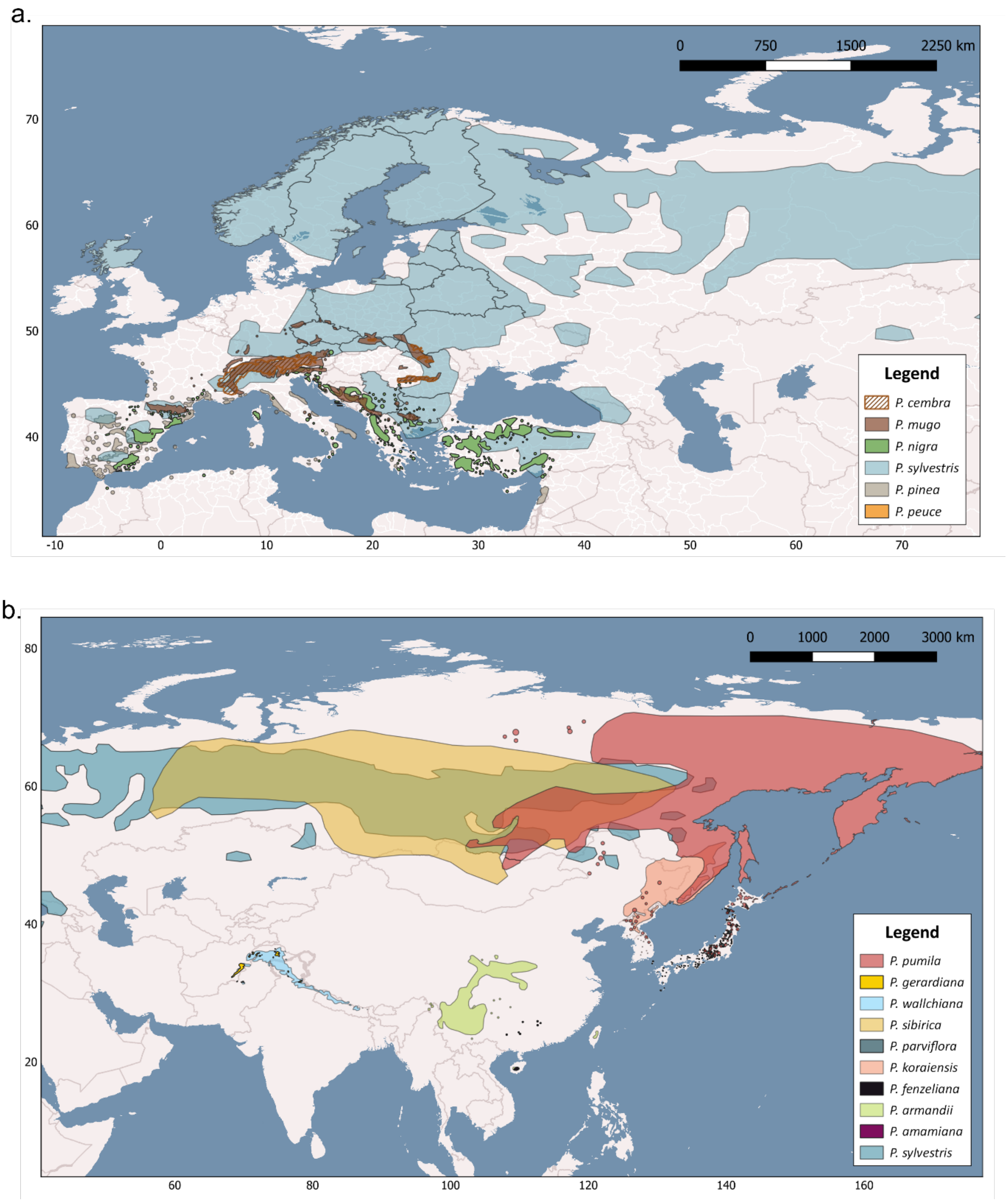

Supplementary Figure 1. Vegetation and distribution maps of plants associated with Nutcrackers. a) European pine (*Pinus*), b) Asian pines, c) Hazelnut (*Corylus*) and d) Spruce (*Picea*). See Supplementary Table 11 for further information.

c.

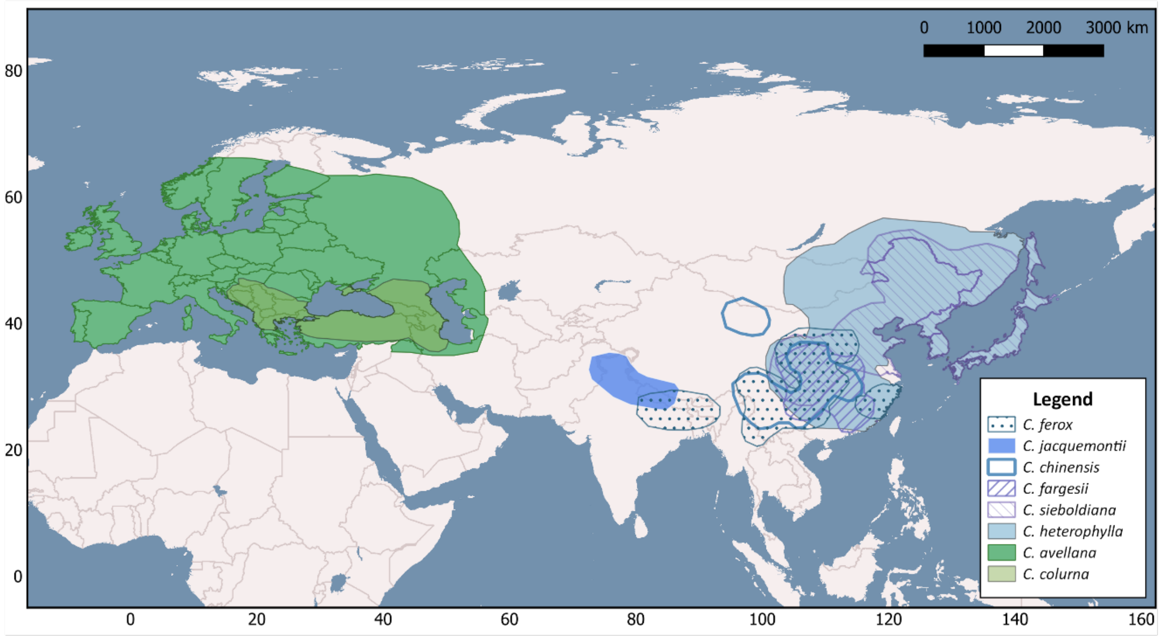

d.

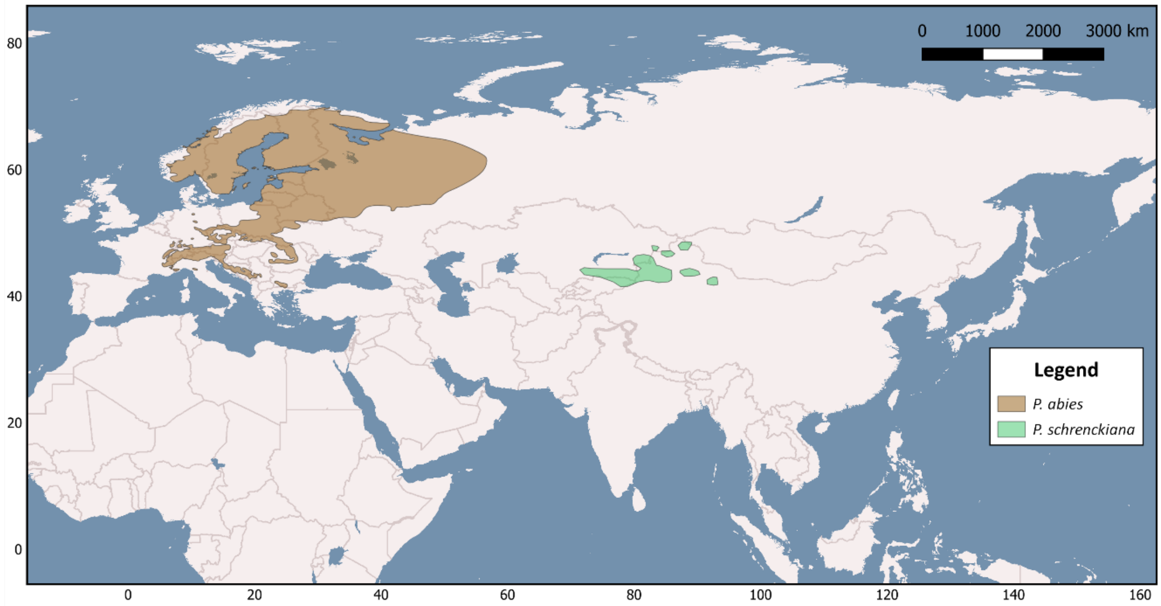

Supplementary Figure 1 (continued). Vegetation and distribution maps of plants associated with Nutcrackers. a) European pine (*Pinus*), b) Asian pines, c) Hazelnut (*Corylus*) and d) Spruce (*Picea*). See Supplementary Table 11 for further information.

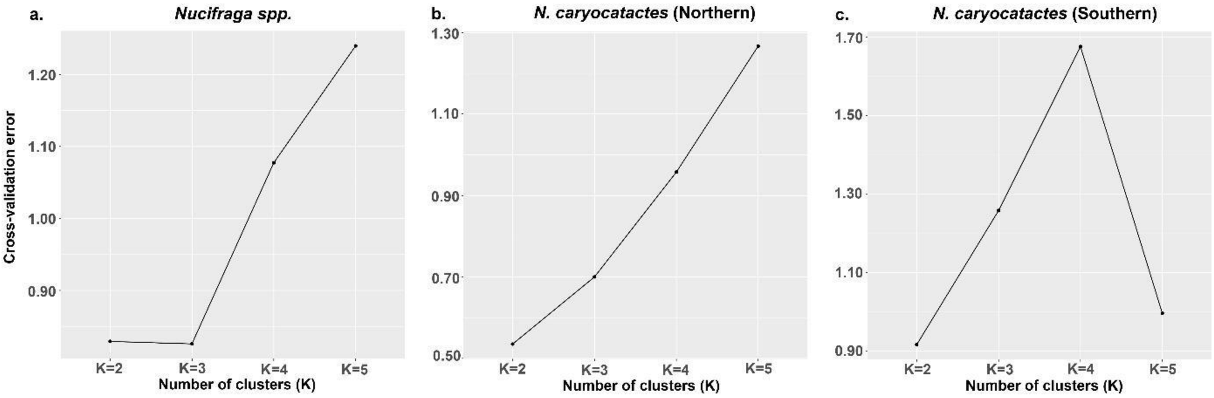

Supplementary Figure 2. Cross-validation error of admixture models a. Admixture analyses of between-species variation identified K=3 as the optimal number of clusters. **b.** Admixture analyses of within-species variation of the Northern *N. caryocatactes* group identified K=2 as the optimal number of clusters. **c.** Admixture analyses of within-species variation of the Southern *N. caryocatactes* group similarly identified K=2 as optimal number of clusters.

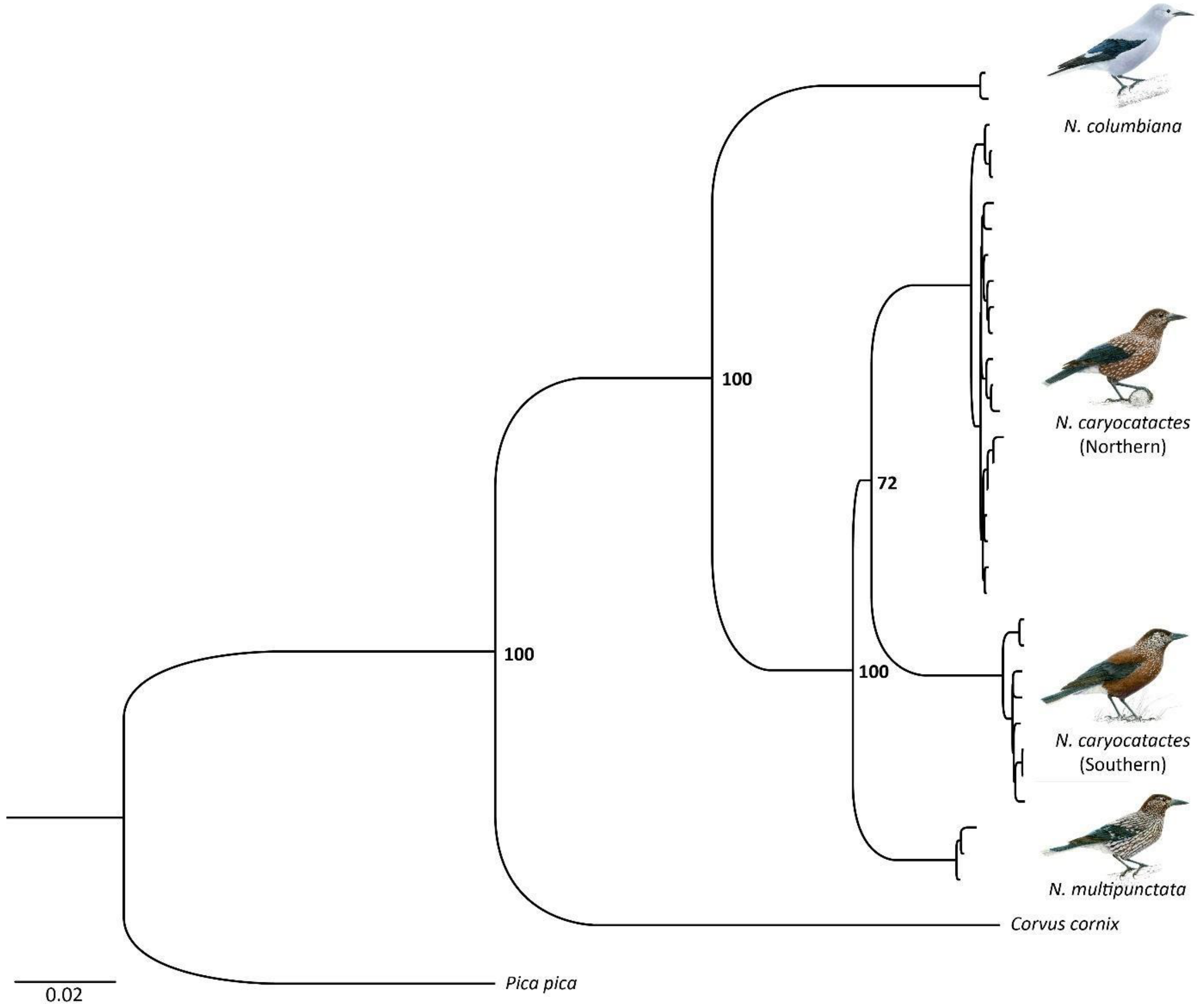

Supplementary Figure 3. Phylogenetic analyses based on mitochondrial genomes. Maximum likelihood tree inference was performed based on a 10,851 bp protein-coding mitochondrial alignment. Numbers at the nodes represent ultrafast bootstrap support. Bird images by Jon Baldur Hlidberg, courtesy of the authors.

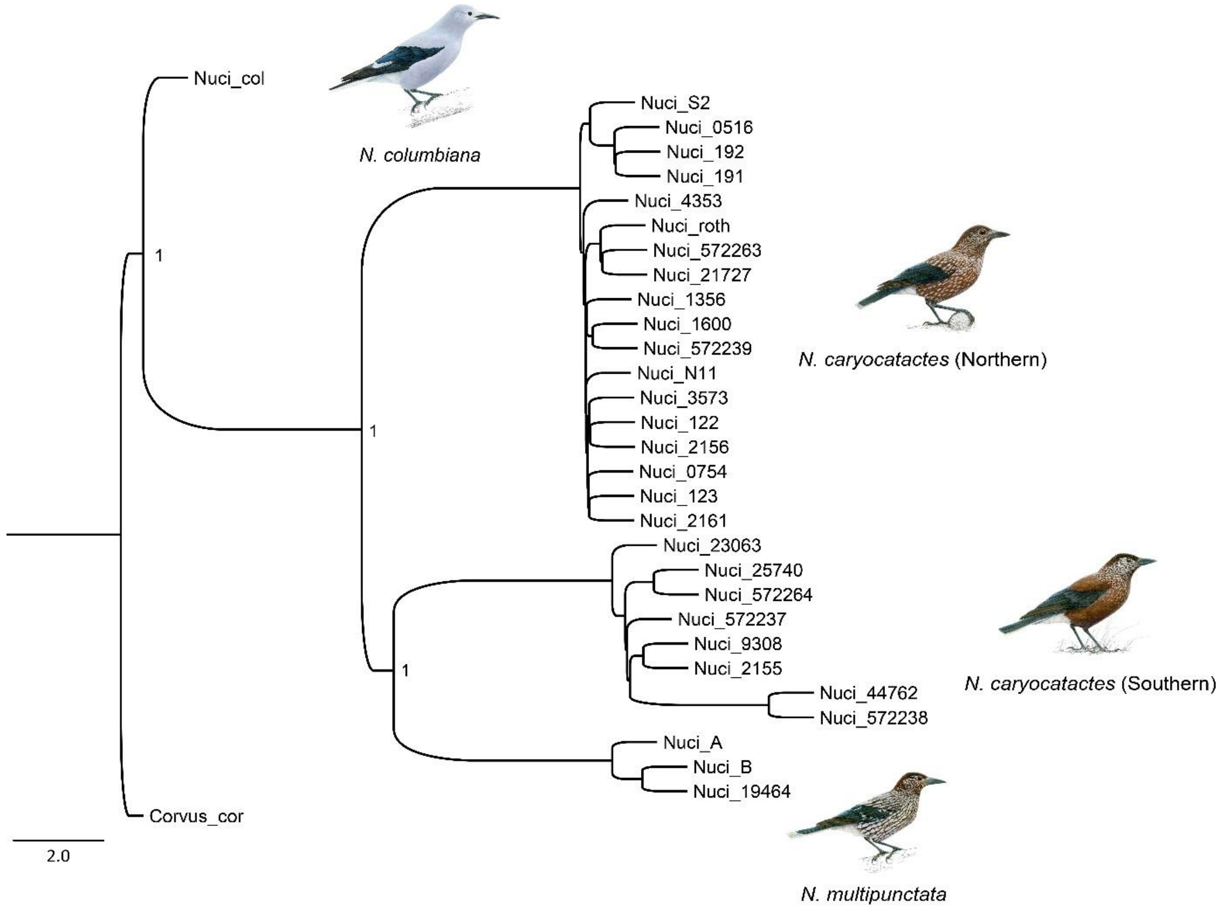

Supplementary Figure 4. Multispecies coalescence tree based on the nuclear genome. Species tree based on the multispecies coalescent model (MSC), using maximum likelihood trees inferred from 192 alignments of 1 Mbp of the nuclear genome. Branch lengths are in coalescence units and numbers at the nodes represent posterior probabilities. Three Eurasian nutcracker groups were identified. The Clark's nutcracker (*N. columbiana*) and Hooded Crow (*Corvus cornix*) were included as outgroups. Bird images by Jon Baldur Hlidberg, courtesy of the authors.

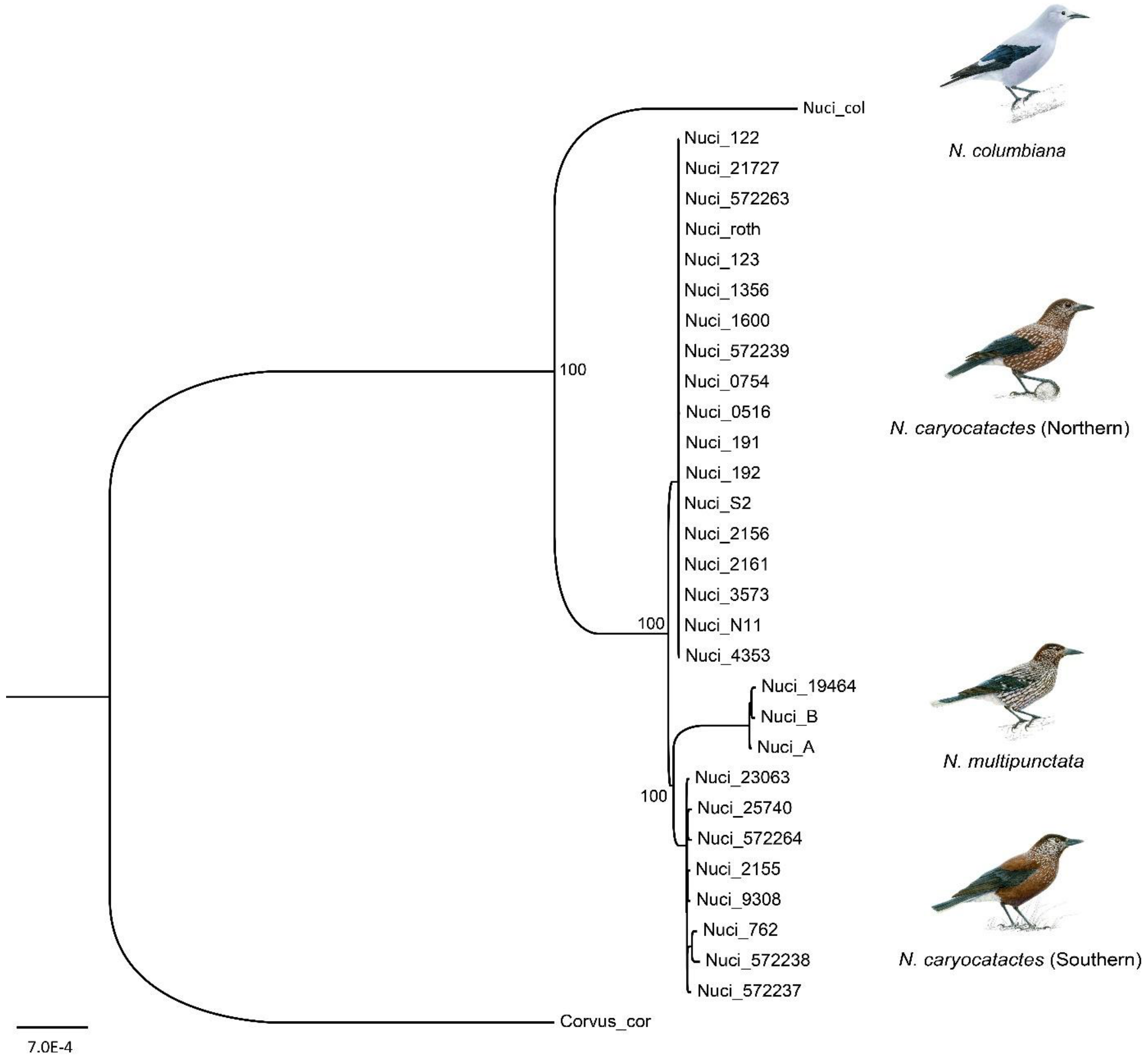

Supplementary Figure 5. Maximum likelihood tree based on the nuclear genome. Maximum likelihood inferences was performed based on the concatenation of 192 alignments of 1 Mbp. Three Eurasian nutcracker lineages were identified, separated by short branches. The Clark's nutcracker (*N. columbiana*) and Hooded Crow (*Corvus cornix*) were included as outgroups. Numbers at the nodes represent bootstrap support. Bird images by Jon Baldur Hlidberg, courtesy of the authors.

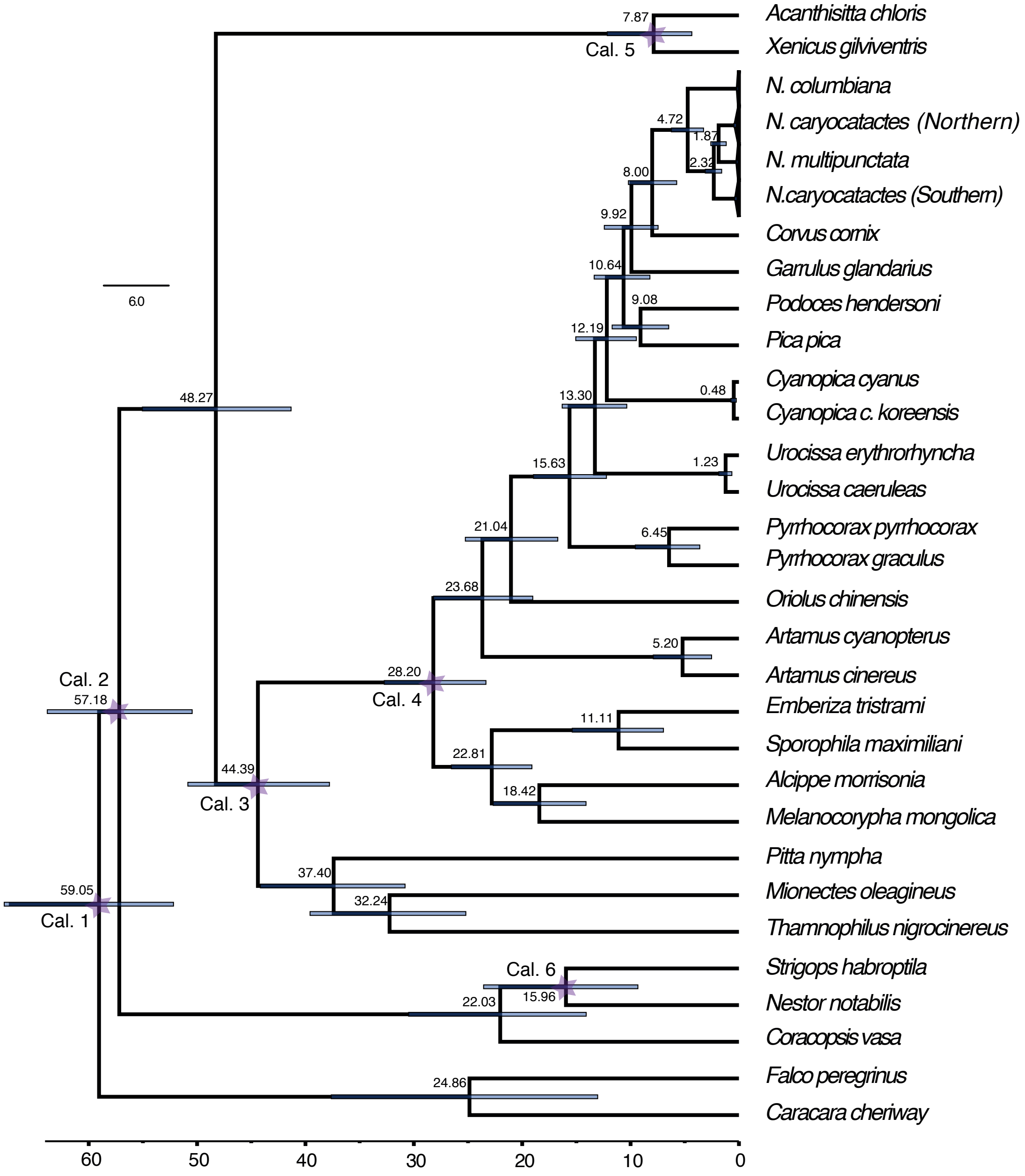

Supplementary Figure 6. Divergence time estimation based on mitochondrial alignments. Divergence time estimates were based on 10,851 bp protein-coding mitochondrial alignments of 59 individuals covering 31 species. The bmodeltest with a transition-transversion split was used to find a fitting substitution model. A relaxed clock log normal and a Birth-Death model was implemented. BEAST prior settings and calibration nodes (Cal. 1-Cal. 6) are provided in Supplementary Table 1. Node ages are in million years. The internal branches are collapsed within *Nucifraga*, and those divergence times are depicted in Supplementary Figure 7.

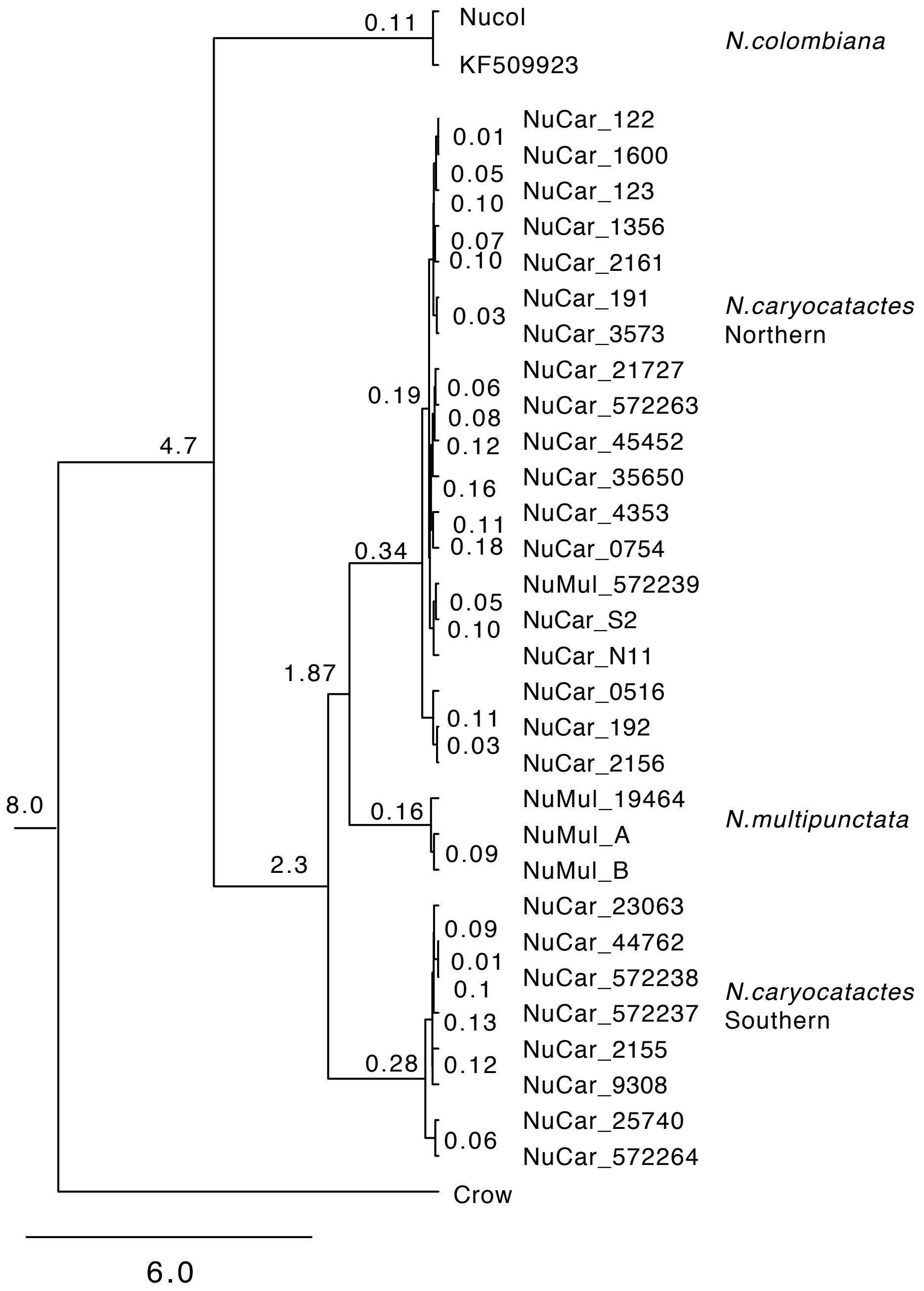

Supplementary Figure 7. Divergence time estimates of the Eurasian nutcracker species complex based on mitochondrial alignments. Divergence times of species and subspecies within *Nucifraga*. Analysis and phylogeny same as Supplementary Figure S6, but displaying the branches for each nutcracker individual. Numbers at the nodes reflect estimated node ages in million years.

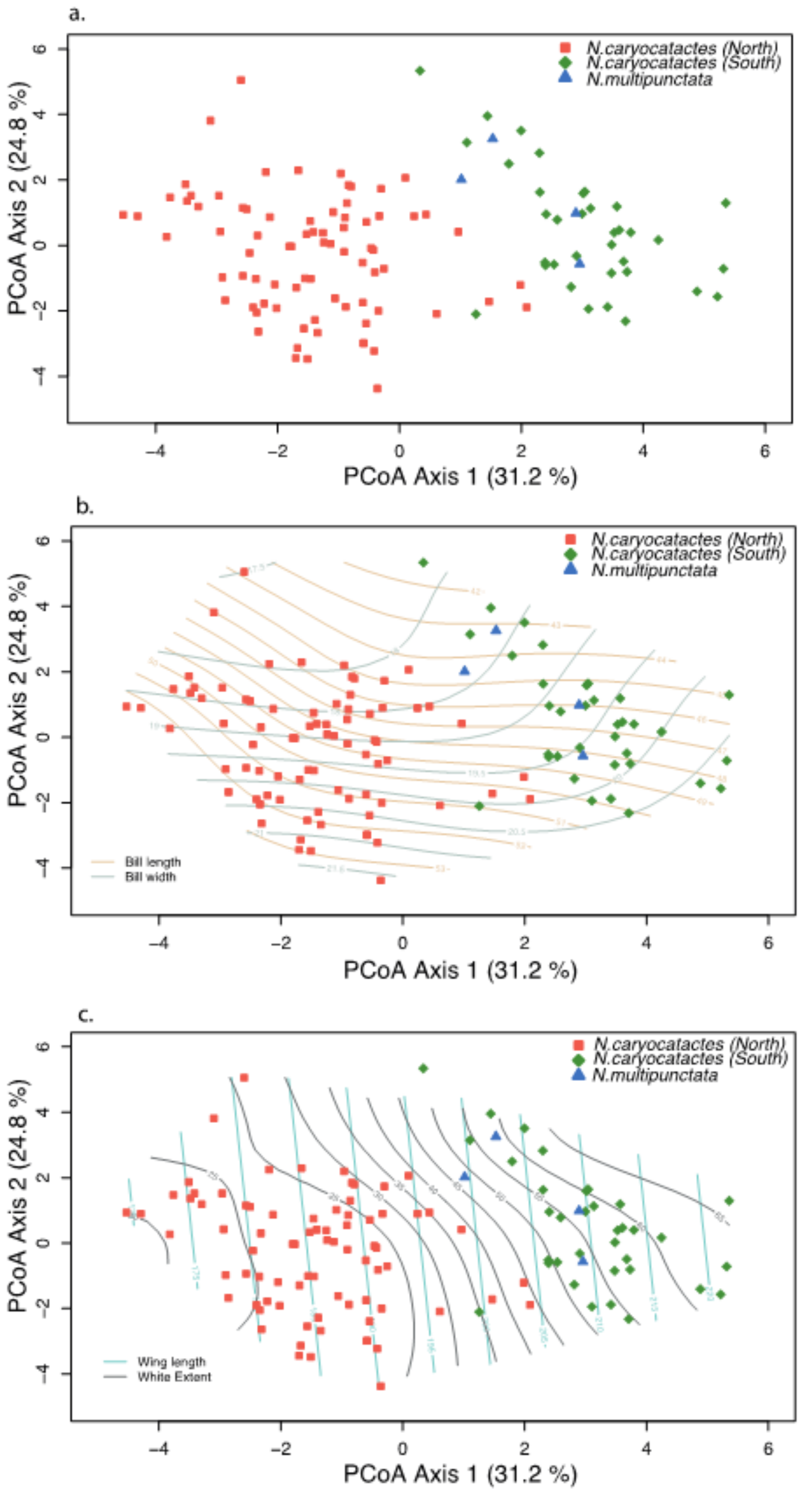

Supplementary Figure 8. Principal coordinate analysis (PCoA) of morphometric traits. a. A PCoA was performed based on 12 morphometric traits (Supplementary Table 10) to examine phenotypic variation in the dataset without a priori defined groups. **b**. Bill morphology did not contribute to the main principal components of the PCoA. **c**. wing length and white extent appear to be the main drivers of phenotypic variation between species.

a.

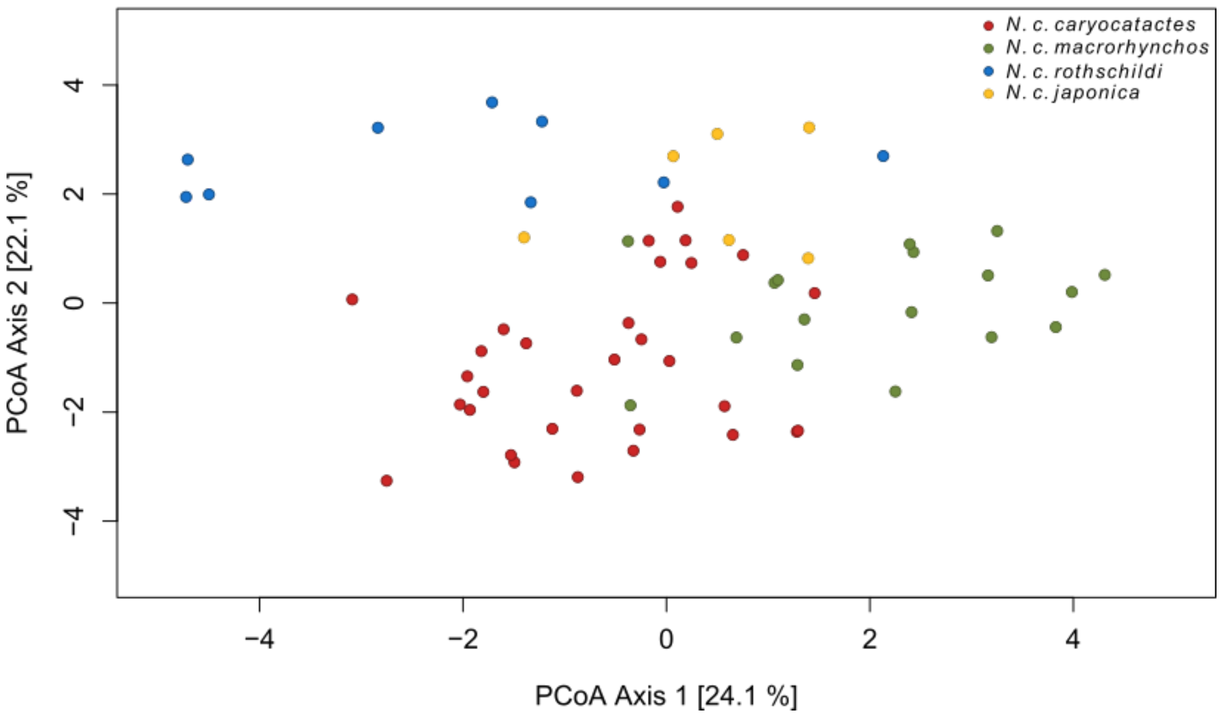

b.

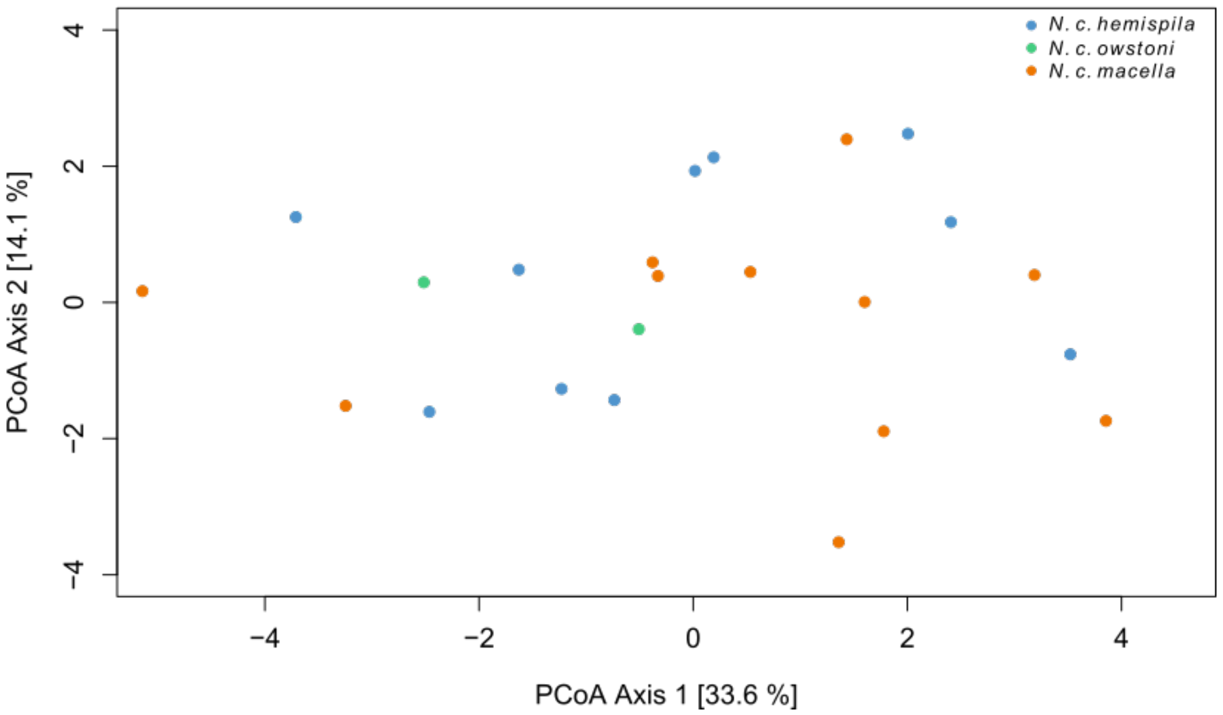

Supplementary Figure 9. Within-species principal coordinate analysis (PCoA) of morphometric traits based on 12 morphometric traits. a. Phenotypic variation within the northern nutcracker subspecies without a priori defined subspecies **b.** Phenotypic variation within the southern nutcracker subspecies without a priori defined subspecies.

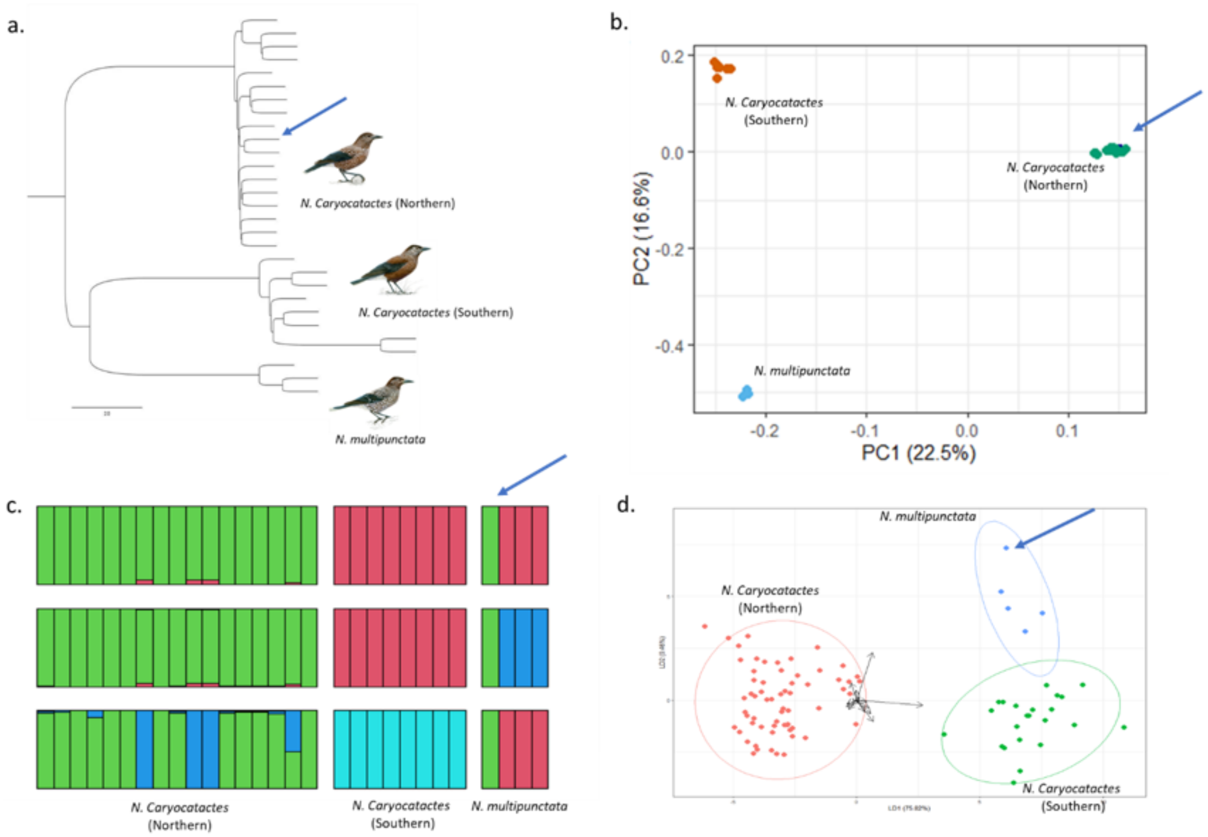

Supplementary Figure 10. Discrepancy between morphology and genetics in one specimen. Within our genomic dataset, one specimen of *N. multipunctata* (Nuci_572239 – indicated with the blue arrow) had a genomic profile similar to those of *N. caryocatactes* (Northern) (**a**-**c**). However, morphological measurements confirm a phenotype similar to those of *N. multipunctata* specimens (**d**). Cross validation of mitochondrial control region based on additional toe-pad material from this specimen confirmed the genetic similarity to *N. caryocatactes* (northern). No clear explanation was identified for this pattern and subsequent analyses were run both including and excluding this specimen. Bird images by Jon Baldur Hlidberg, courtesy of the authors.

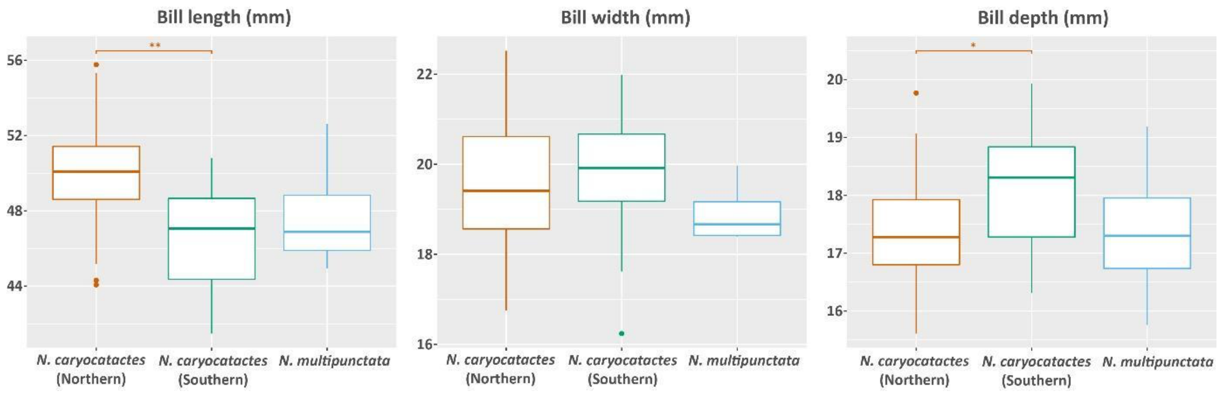

Supplementary Figure 11. Phenotypic variation in beak morphology between nutcracker species. Beak morphology was compared between the northern *N. caryocatactes* (N=63) group, southern *N. caryocatactes* group (N=23) and *N. multipunctata* (N=4) based on an independent morphometric dataset consisting of 90 measured museum specimens. * = significant variation between means. ** = significant variation between means after Bonferroni correction.

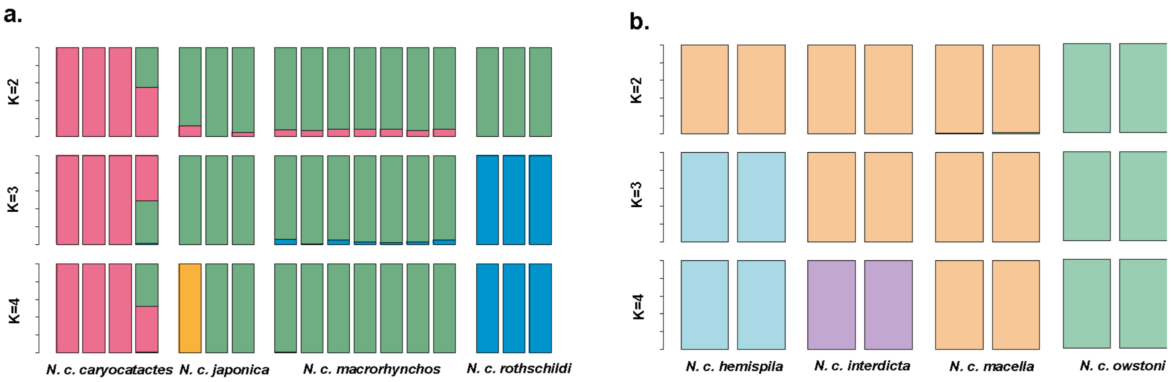

Supplementary Figure 12. Within-species genetic variation based on admixture analyses. a. Admixture analyses of the northern *N. caryocatactes* group (142,799 linkage-pruned SNPs). Each bar represents one individual. One potential F1-hybrid was identified between *N. c. caryocatactes* and *N. c. macrorhynchos* **b.** Within-species genetic variation in the southern *N. caryocatactes* group (101,611 linkage pruned SNPs). Admixture analyses for both the northern and southern nutcracker species had the lowest cross-validation error at K=2 (Supplementary Figure 2).

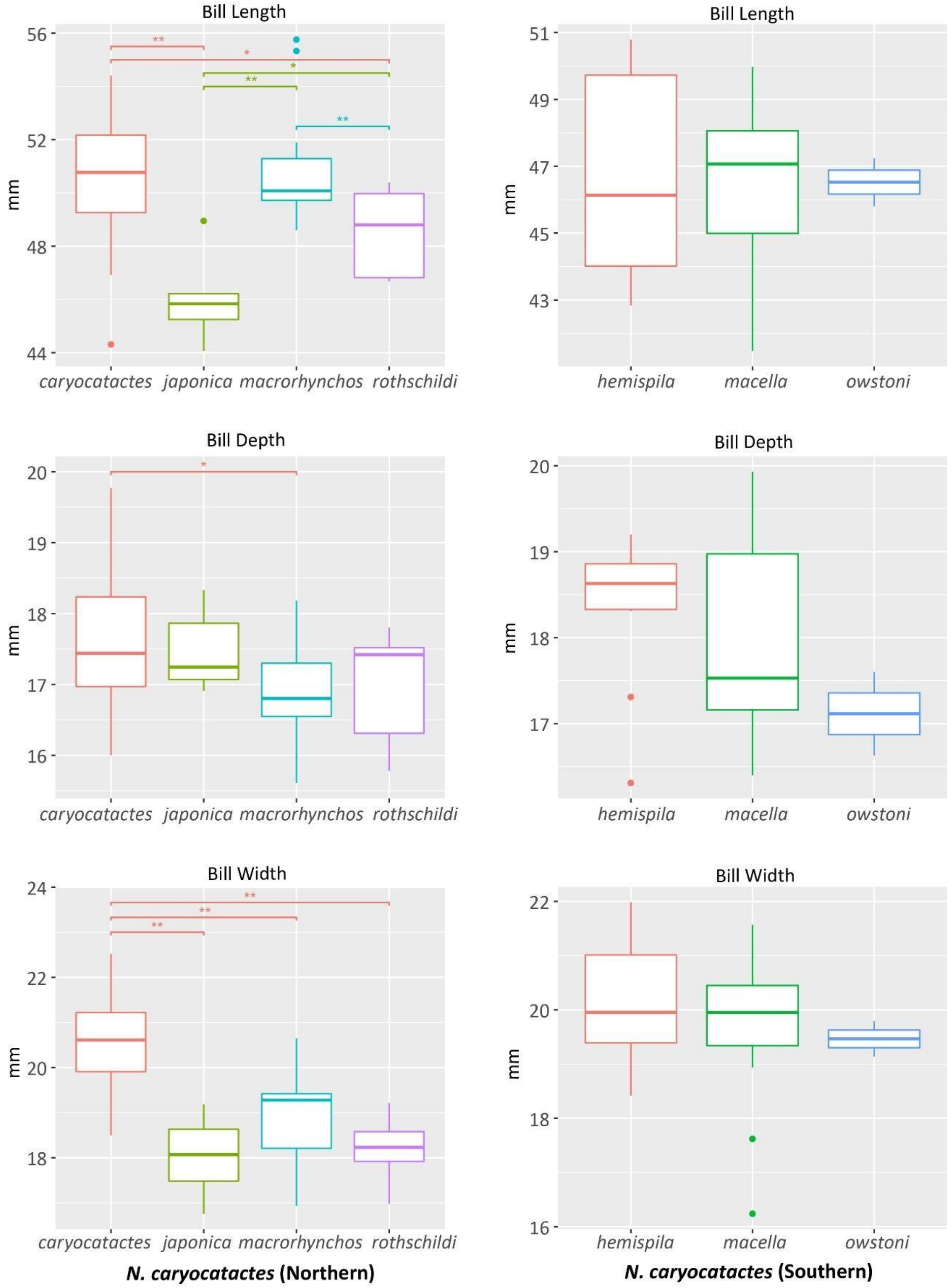

Supplementary Figure 13. Phenotypic variation in beak morphology between subspecies. Morphometric measurements of beak morphology per subspecies. *N. c. caryocatactes* (N=31), *N. c. macrorhynchos* (N=17), *N. c. rothschildi* (N=9), *N. c. japonica* (N=6), *N. c. hemispila* (N=11), *N. c. macella* (N=10) and *N. c. owstoni* (N=2). * = Significant variation between means. ** = significant variation between means after Bonferroni correction.

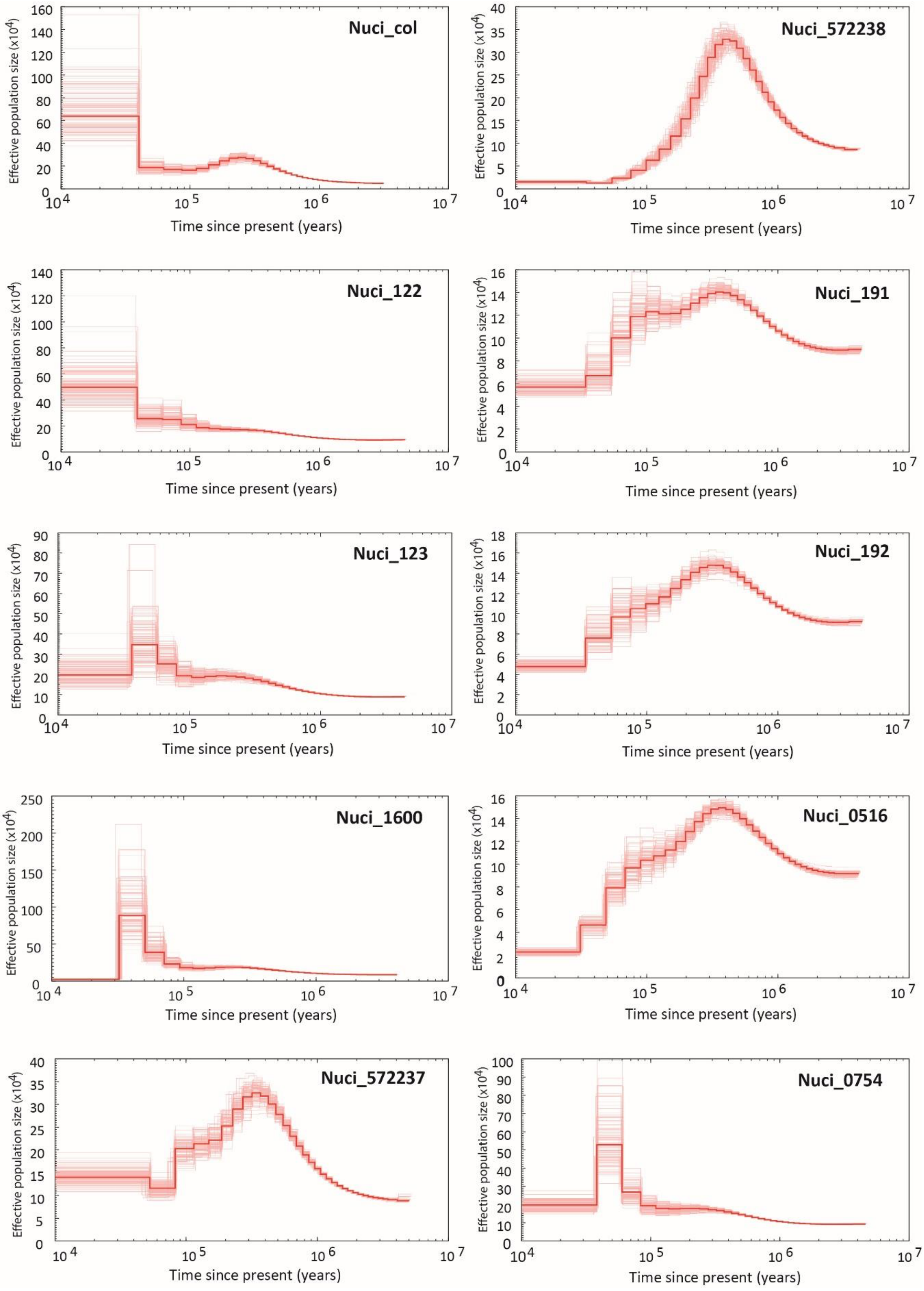

Supplementary Figure 14. Reconstruction of demographic history (PSMC). PSMC plots visualized per individual. Only those individuals with a minimum coverage of 17X are included, as recommended in Nadachowska-Brzyska et al.[1]. PSMC was run for 25 iterations with the upper limit of the TMRCA set to -t 5, the initial h/q value to -r 1, and 34 atomic time intervals (4+30*2+4+6+10). A mutation rate (μ) of 3.18×10^{-9} substitutions per generation was used[2] and a generation time (g) of 7 years[3].

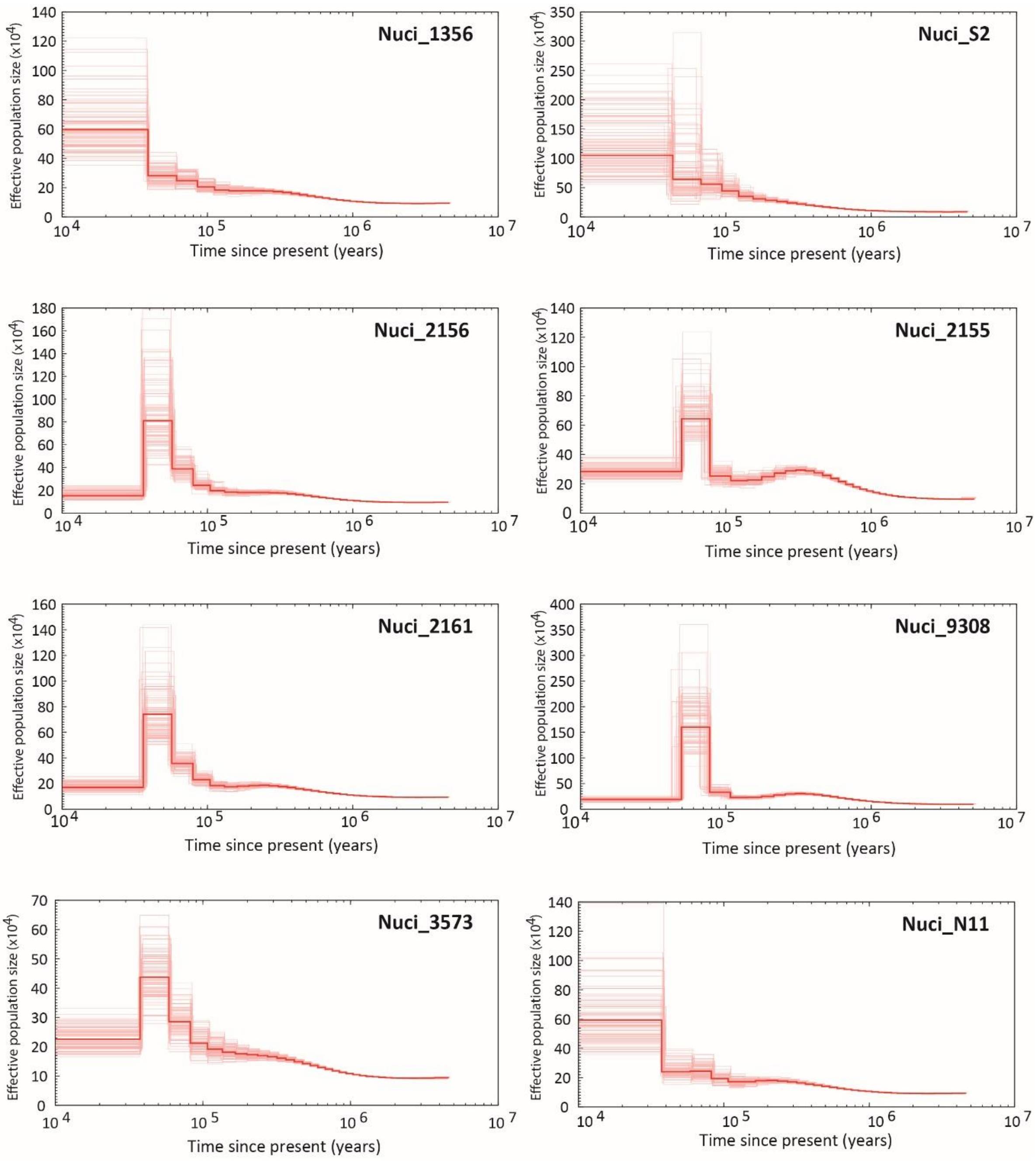

Supplementary Figure 14 (continued). Reconstruction of demographic history (PSMC). PSMC plots visualized per individual. Only those individuals with a minimum coverage of 17X are included, as recommended in Nadachowska-Brzyska et al.[1] PSMC was run for 25 iterations with the upper limit of the TMRCA set to -t 5, the initial h/q value to -r 1, and 34 atomic time intervals (4+30*2+4+6+10). A mutation rate (μ) of 3.18×10^{-9} substitutions per generation was used[2] and a generation time (g) of 7 years[3].

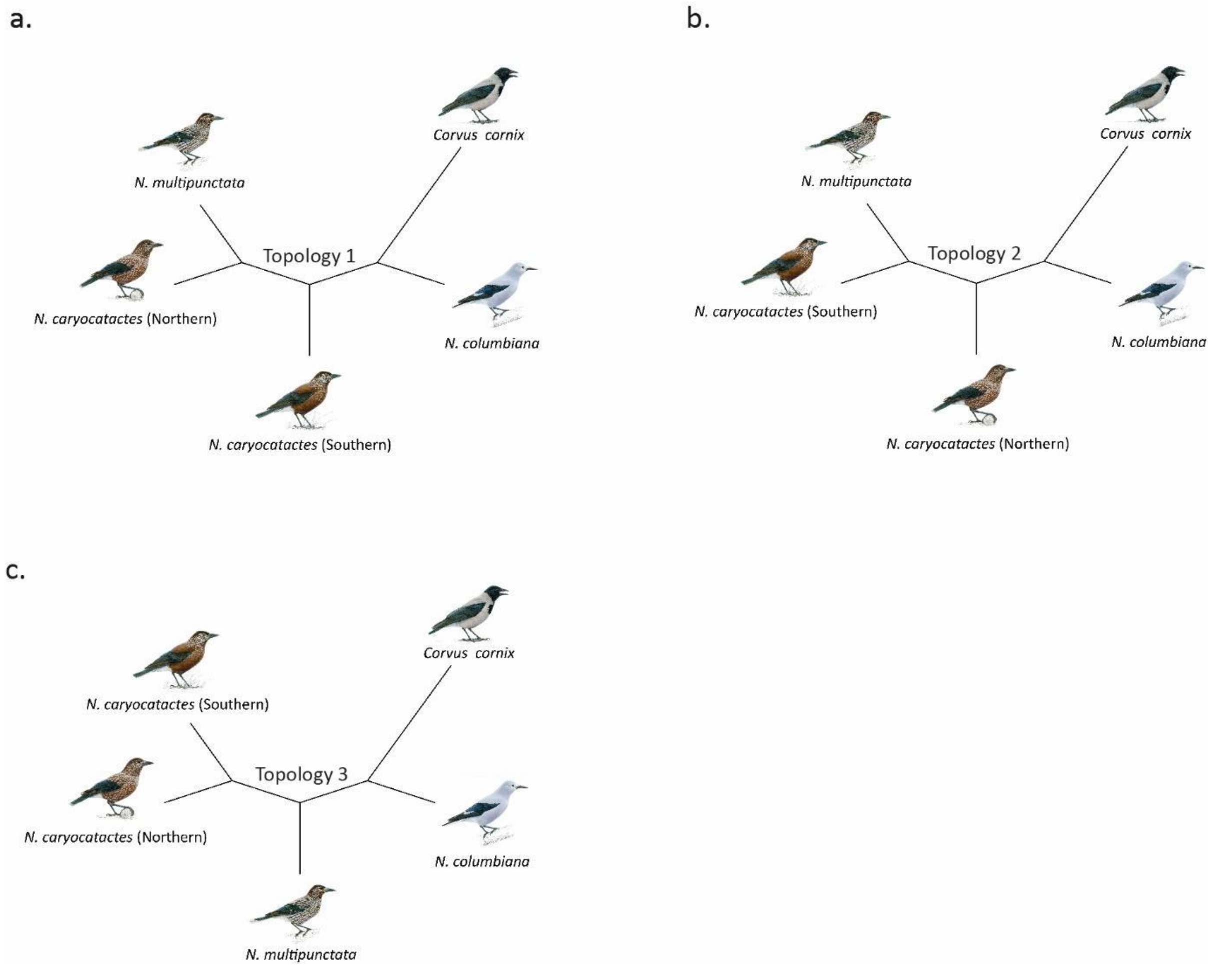

Supplementary Figure 15. Possible topologies for the nutcracker species complex. **a.** *N. multipunctata* and *N. caryocatactes* (Northern) form a sister group to *N. caryocatactes* (Southern). **b.** *N. multipunctata* and *N. caryocatactes* (Southern) form a sister group to *N. caryocatactes* (Northern). **c.** Both the northern and southern *N. caryocatactes* groups form a sister group to *N. multipunctata*. Topologies were evaluated with the approximate unbiased test (AU-test) to determine whether alternative topologies could be rejected (Supplementary Figure 16). Bird images by Jon Baldur Hlidberg, courtesy of the authors.

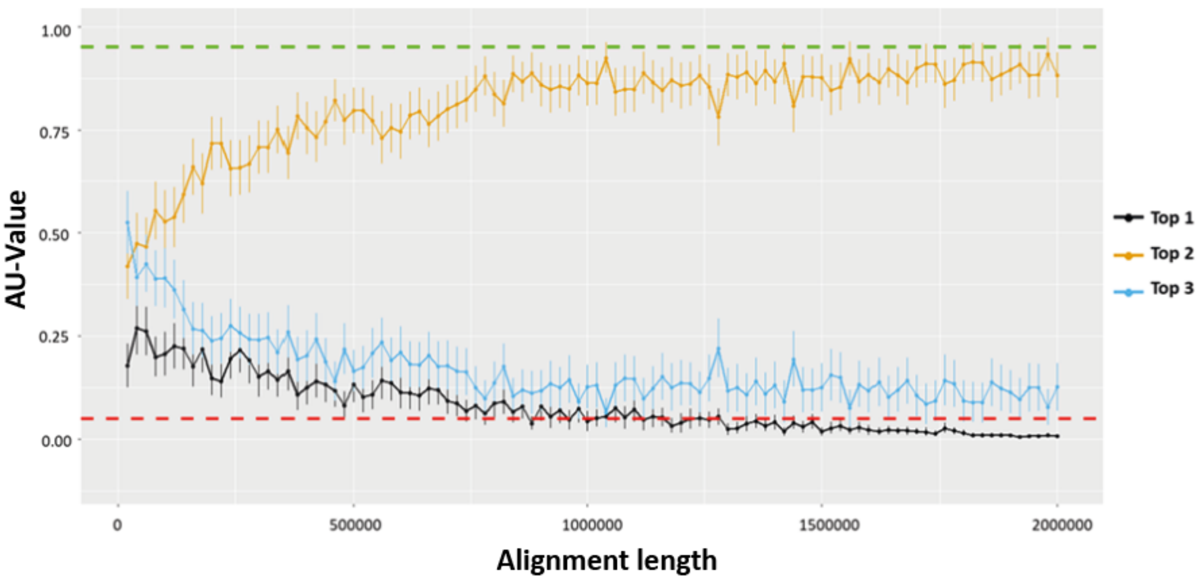

Supplementary Figure 16. **Approximate unbiased test for phylogenetic inference**. An approximate unbiased test was performed on increasing genome alignment lengths to infer the minimum alignment length needed for accurate phylogenetic inference. The X-axis represents the increase in window size whereas the Y-axis shows the corresponding mean AU-score for the window length. Increasing the window length reveals that topology 1 (black line, Supplementary Figure 15a) could significantly be rejected at an alignment length of >1 Mbp, reaching an AU-value of < 0.05. The second topology (Supplementary Figure 15b) seems to be the most likely (orange line), but never reaches the significant threshold of >0.95.

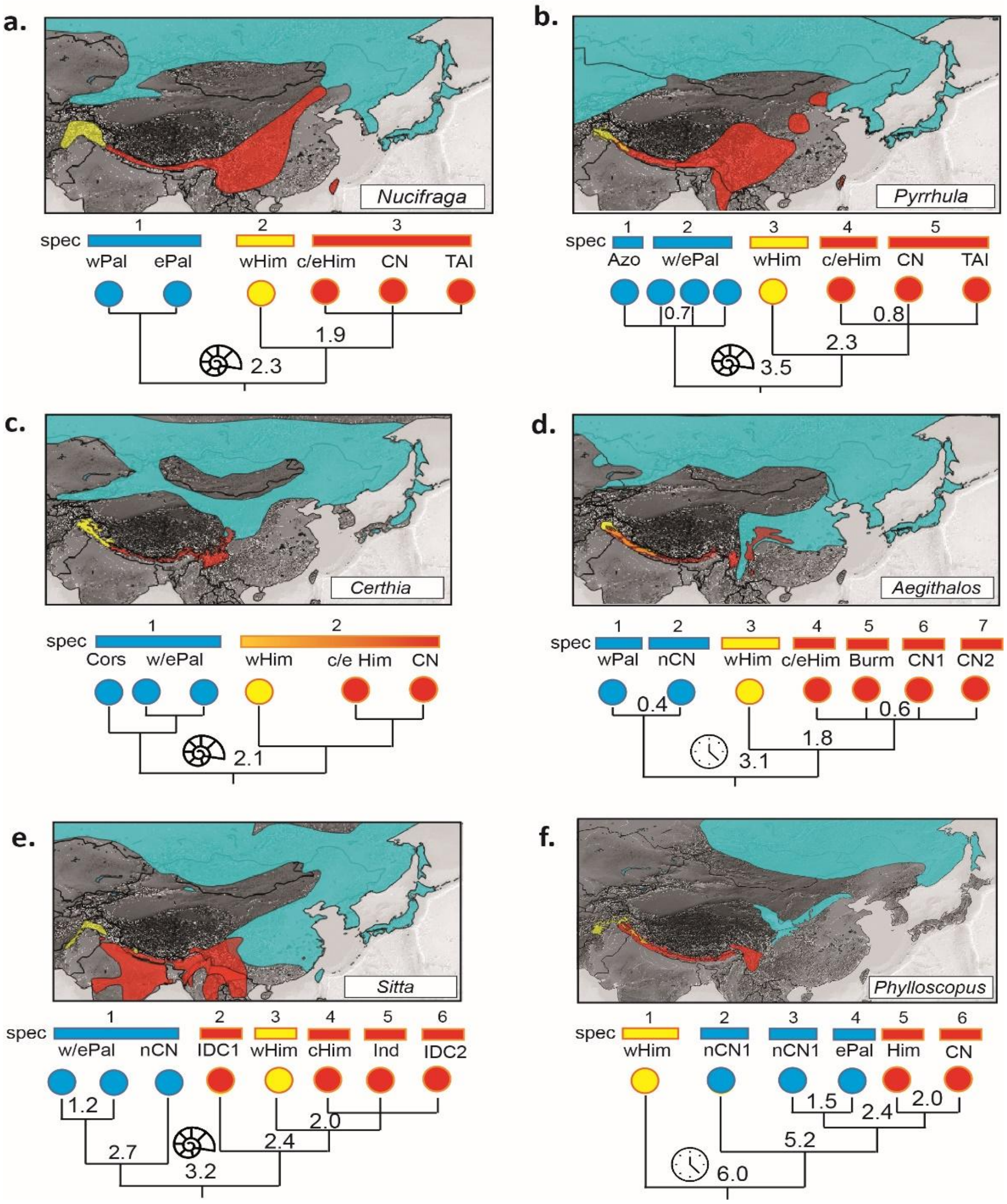

Supplementary Figure 17. Similarity in phylogeographic and divergence patterns of Sino-Himalayan passerines. Comparison of the nutcracker diversification compared to other non-mutualistic Sino-Himalayan passerine birds occurring in the same region. **a.** nutcrackers, *Nucifraga*: 1= *N. caryocatactes*, 2= *N. hemispila*, 3= *N. multipunctata*; **b.** bullfinches, *Pyrrhula*: 1= *P. murina*, 2= *P. pyrrhula*, 3= *P. aurantiaca*, 4= *P. erythrocephala*, 5= *P. erythaca*[4] **c.** treecreepers, *Certhia*: 1= *Certhia familiaris*, 2= *Certhia hodgsoni*[5] **d.** bushtits, *Aegithalos*: 1= *Ae. caudatus*, 2= *Ae. glaucogularis*, 3= *Ae. niveogularis*, 4= *Ae. iouschistos*, 5= *Ae. sharpei*, 6= *Ae. bonvaloti*, 7= *Ae. fuliginosus*[5] **e.** nuthatches, *Sitta*: 1= *S. europaea*, 2= *S. nagaensis*, 3= *S. cashmirensis*, 4= *S. cinnamoventris*, 5= *S. castanea*, 6= *S. neglecta*[6] **f.** leaf warblers, *Phylloscopus*: 1= *P. subviridis*, 2= *P. yunnanensis*, 3= *P. kansuensis*, 4= *P. proregulus*, 5= *P. chloronotus*, 6= *P. forresti*[7]. Mean divergence time estimates (in Myr) indicated at nodes. Shells indicate use of fossils in calibration whereas clocks indicate fixed mtDNA rates. Distribution maps according to BirdLife International[3]. Abbreviations: n/e/s/w= northern/eastern/southern/western, Pal= Palearctic, Azo=Azores, Cors= Corsica, CN= China, Him= Himalayas, Burm= Myanmar, Ind= India, IDC= Indochina, TAI= Taiwan.

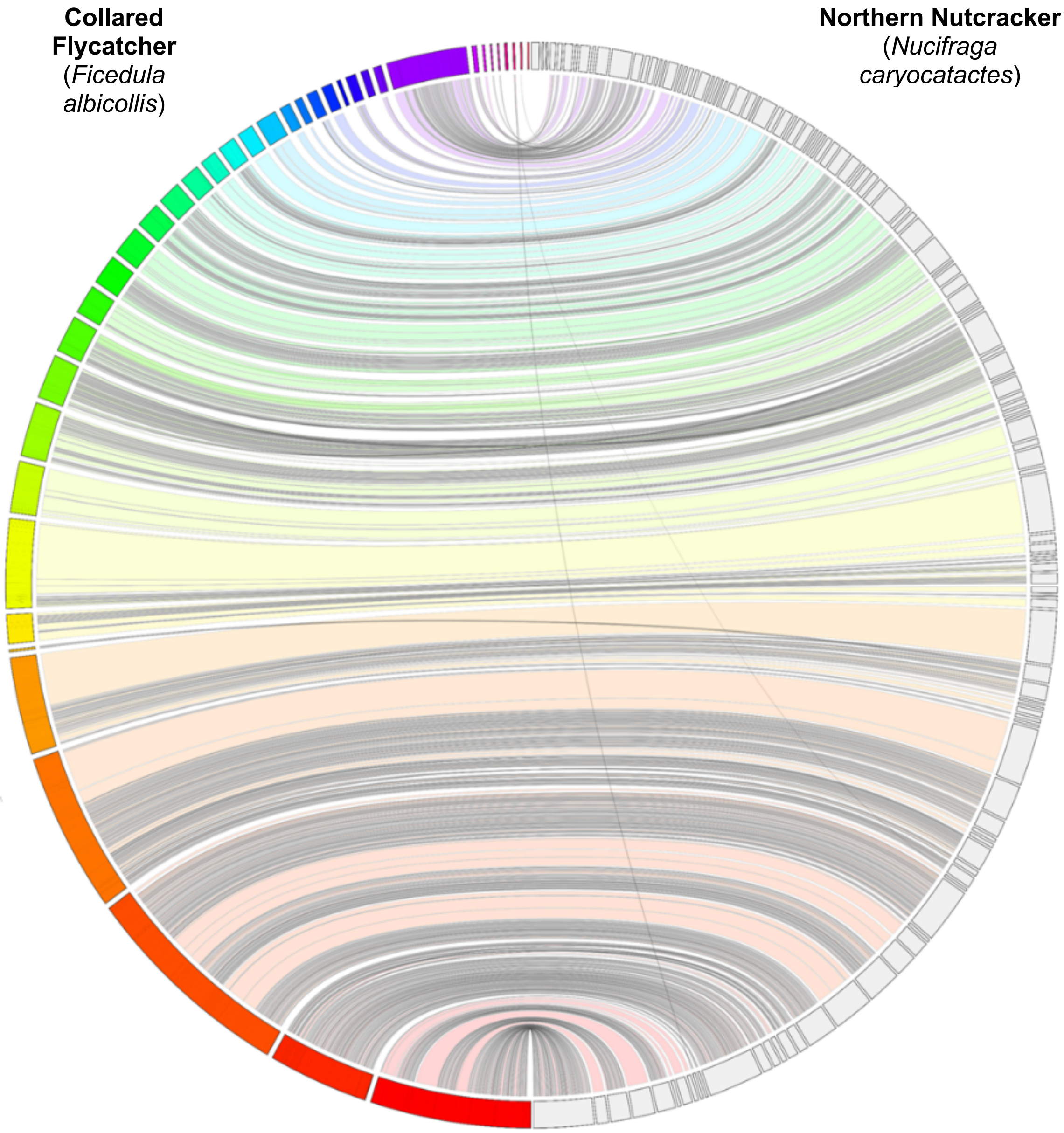

Supplementary Figure 18. Synteny between the assembled nutcracker genome and chromosome level flycatcher genome. Synteny plot visualizing the concordance between the assembled *Nucifraga caryocatactes* genome (right) and the chromosome level Collared Flycatcher (*Ficedula albicollis*) genome (left).

Supplementary Table 1. Calibration points used for molecular dating and tMRCA prior settings in BEAST. Five lognormal priors were included based on previously published fossil and divergence data. Prior mean and standard deviation were set to cover the interval of the fossil age range.

Calibration node	node dated	type	date (Ma)	offset	mean	sd	reference
1	Root (Falconiformes)	Normal	52-62	0.0	59.1	3.8	Oliveros et al.[8]
2	Psittaciformes vs Passeriformes	normal	51.81-66.5	0.0	41	13.0	Oliveros et al.[8]
3	oscines vs suboscines	normal	27.52-56.0	0.0	42.0	8.0	Oliveros et al.[8]
4	Corvides vs Passerides	normal	17–26	0.0	21	2.45	Jarvis et al.[9]
5	*Acanthisitta* vs *Xenicus*	normal	15.9-56.0	0.0	36	10.2	Oliveros et al.[8]

Supplementary Table 2. Measurements and comparisons of morphometric traits for the three main *Nucifraga* groups. Summary statistics of the primary traits identified in the LDA are shown on the left, whereas statistical comparisons using Welch's t-test are presented on the right. Double asterixis represent significant variation after Bonferroni correction ($p \leq 0.008$).

**Wing length**				**Welch's t-test**			
**Species**	**n**	**mean**	**st.dev**		*N. caryocatactes* (Northern)	*N. caryocatactes* (Southern)	*N. multipunctata*
N. caryocatactes (Northern)	63	185.64	8.49	*N. caryocatactes* (Northern)	-		
N. caryocatactes (Southern)	23	210.27	8.91	*N. caryocatactes* (Southern)	**	-	
N. multipunctata	4	211.52	5.20	*N. multipunctata*	**	n.s.	-
**Tail length**				**Welch's t-test**			
**Species**	**n**	**mean**	**st.dev**		*N. caryocatactes* (Northern)	*N. caryocatactes* (Southern)	*N. multipunctata*
N. caryocatactes (Northern)	63	124.38	6.15	*N. caryocatactes* (Northern)	-		
N. caryocatactes (Southern)	23	140.12	7.42	*N. caryocatactes* (Southern)	**	-	
N. multipunctata	4	155.63	6.13	*N. multipunctata*	**	**	-
**White extent**				**Welch's t-test**			
**Species**	**n**	**mean**	**st.dev**		*N. caryocatactes* (Northern)	*N. caryocatactes* (Southern)	*N. multipunctata*
N. caryocatactes (Northern)	63	23.54	4.17	*N. caryocatactes* (Northern)	-		
N. caryocatactes (Southern)	23	59.22	9.51	*N. caryocatactes* (Southern)	**	-	
N. multipunctata	4	56.37	5.20	*N. multipunctata*	**	n.s.	-

Supplementary Table 3. Measurements and comparisons of morphometric traits within the Northern *N. caryocatactes* group. Summary statistics of the primary traits identified in the LDA are shown on the left, whereas statistical comparisons using Welch's t-test are presented on the right. Asterixis represents significant variation ($p \leq 0.05$), whereas double asterixis represent significant variation after Bonferroni correction ($p \leq 0.008$).

**Wing length**					**Welch's t-test**				
**Subspecies**	**n**	**mean**	**st.dev**			*N. c. caryocatactes*	*N. c. macrorhynchos*	*N. c. rothschildi*	*N. c. japonica*
N. c. caryocatactes	31	184.57	5.24		*N. c. caryocatactes*	-			
N. c. macrorhynchos	17	179.4	4.99		*N. c. macrorhynchos*	**	-		
N. c. rothschildi	9	200.23	4.8		*N. c. rothschildi*	**	**	-	
N. c. japonica	6	187	9.41		*N. c. japonica*	n.s.	n.s.	*	-
**White extent**					**Welch's t-test**				
**Subspecies**	**n**	**mean**	**st.dev**			*N. c. caryocatactes*	*N. c. macrorhynchos*	*N. c. rothschildi*	*N. c. japonica*
N. c. caryocatactes	31	21.82	3.55		*N. c. caryocatactes*	-			
N. c. macrorhynchos	17	26.71	4.27		*N. c. macrorhynchos*	**	-		
N. c. rothschildi	9	23.05	2.55		*N. c. rothschildi*	n.s.	*	-	
N. c. japonica	6	24.25	4.26		*N. c. japonica*	n.s.	n.s.	n.s.	-
**Tail length**					**Welch's t-test**				
**Subspecies**	**n**	**mean**	**st.dev**			*N. c. caryocatactes*	*N. c. macrorhynchos*	*N. c. rothschildi*	*N. c. japonica*
N. c. caryocatactes	31	122.52	3.56		*N. c. caryocatactes*	-			
N. c. macrorhynchos	17	120.76	3.12		*N. c. macrorhynchos*	n.s.	-		
N. c. rothschildi	9	135.94	4.79		*N. c. rothschildi*	**	**	-	
N. c. japonica	6	127.00	3.22		*N. c. japonica*	*	**	**	-

Supplementary Table 4. Measurements and comparisons of morphometric traits within the Southern *N. caryocatactes* group. Summary statistics are shown on the left, whereas statistical comparisons using Welch's t-test are presented on the right. Statistical testing for the *N. c. owstoni* subspecies could not be performed due to the small sample size (n=2).

**Wing length**					**Welch's t-test**			
**Subspecies**	**n**	**mean**	**st.dev**			*N. c. macella*	*N. c. hemispila*	*N. c. owstoni*
N. c. macella	11	214.18	9.75		*N. c. macella*	-		
N. c. hemispila	10	207.77	6.45		*N. c. hemispila*	n.s.	-	
N. c. owstoni	2	201.25	5.30		*N. c. owstoni*	N/A	N/A	-
**Distal distance**					**Welch's t-test**			
**Subspecies**	**n**	**mean**	**st.dev**			*N. c. macella*	*N. c. hemispila*	*N. c. owstoni*
N. c. macella	11	88.95	7.74		*N. c. macella*	-		
N. c. hemispila	10	85.5	5.7		*N. c. hemispila*	n.s.	-	
N. c. owstoni	2	82.5	0.70		*N. c. owstoni*	N/A	N/A	-
**Bill length**					**Welch's t-test**			
**Subspecies**	**n**	**mean**	**st.dev**			*N. c. macella*	*N. c. hemispila*	*N. c. owstoni*
N. c. macella	11	46.35	2.66		*N. c. macella*	-		
N. c. hemispila	10	46.75	3.18		*N. c. hemispila*	n.s.	-	
N. c. owstoni	2	46.52	1.00		*N. c. owstoni*	N/A	N/A	-

Supplementary Table 5. ***Nucifraga*** **genome assembly summary statistics**

Assembly statistic	*Nucifraga*
**Number of contigs/scaffolds**	12,498
**Assembly length**	1,144,305,354
**Number of contigs/scaffolds (> 100 kbp)**	294
**Assembly length (> 100kbp)**	1,076,014,238
**Largest scaffold**	45.16 Mbp
**Contig N50**	217.12 Kbp
**Scaffold N50**	12.61 Mbp
**Scaffold L50**	24

Supplementary Table 6. Repeat content of the de novo assembled *Nucifraga* genome.

TE	Copy Number	Length	Percentage of assembly
**SINEs**	7,762	914,050	0.08%
**LINEs**	157,615	38,193,048	3.55%
**LTR elements**	83,482	45,888,286	4.26%
**DNA transposons**	5,761	779,916	0.07%
**Unclassified**	12,519	2,176,130	0.20%
**Small RNA**	598	57,527	0.00%
**Satellites**	2,227	715,425	0.07%
**Simple repeats**	207,429	8,726,825	0.81%
**Low complexity**	42,608	2,166,229	0.20%
		**Total:**	9.23%

Supplementary Table 7. Sample collection information. Abbreviations of collections refer to: **ZMMU** = Zoological Museum of Moscow University, **NRM** = Natural History Museum of Sweden, **OLML** = Oberösterreichisches Landesmuseum of Linz , **YIO** = Yamashina Institute for Ornithology, **SNSD** = Senckenberg Natural History Collections of Dresden, **NHMO** = Natural History Museum of Norway, **NHMD** = Natural History Museum of Denmark, **MFNB** = Museum für Naturkunde Berlin, **RAM** = Royal Alberta Museum.

Genus	Species	Subspecies	Collection	sample ID	Voucher No.	Sample type	Sampling location
Nucifraga	*caryocatactes*	*caryocatactes*	ZMMU	Nuci_0516	MVK 430	Tissue	Moscow obl.
Nucifraga	*caryocatactes*	*caryocatactes*	NRM	Nuci_S2	976416	Tissue	Björkö-Arholma
Nucifraga	*caryocatactes*	*caryocatactes*	OLML	Nuci_191	OLML 2017/191	Tissue	Steiermark Möderbrugg
Nucifraga	*caryocatactes*	*caryocatactes*	OLML	Nuci_192	OLML 2017/192	Tissue	Oberösterreich Windischgarsten
Nucifraga	*caryocatactes*	*japonica*	YIO	Nuci_1600	2017-1600	Muscle	Hokkaido
Nucifraga	*caryocatactes*	*japonica*	SNSD	Nuci_4353	MTD-C4353	Skin	Honshu Island
Nucifraga	*caryocatactes*	*japonica*	ZMMU	Nuci_0754	RYA 1560	Tissue	Kuril Island
Nucifraga	*caryocatactes*	*macrorhynchos*	ZMMU	Nuci_3573	RYA 3573	Tissue	Kamchatka
Nucifraga	*caryocatactes*	*macrorhynchos*	NHMO	Nuci_N11	NHMO-BI-39155/1-B	Blood	Björkö-Arholma
Nucifraga	*caryocatactes*	*macrorhynchos*	ZMMU	Nuci_1356	RYA 2655	Tissue	Sakhalin
Nucifraga	*caryocatactes*	*macrorhynchos*	ZMMU	Nuci_2161	SAP 624	Tissue	Kaliningrad obl.
Nucifraga	*caryocatactes*	*macrorhynchos*	ZMMU	Nuci_2156	NIA 629	Tissue	Krasnodar krai
Nucifraga	*caryocatactes*	*macrorhynchos*	NHMD	Nuci_122	116122	Tissue	Altay Mountain Region
Nucifraga	*caryocatactes*	*macrorhynchos*	NHMD	Nuci_123	116123	Tissue	Altay Mountain Region

Supplementary Table 7 (continued). Sample collection information. Abbreviations of collections refer to: **ZMMU** = Zoological Museum of Moscow University, **NRM** = Natural History Museum of Sweden, **OLML** = Oberösterreichisches Landesmuseum of Linz , **YIO** = Yamashina Institute for Ornithology, **SNSD** = Senckenberg Natural History Collections of Dresden, **NHMO** = Natural History Museum of Norway, **NHMD** = Natural History Museum of Denmark, **MFNB** = Museum für Naturkunde Berlin, **RAM** = Royal Alberta Museum.

Genus	Species	Subspecies	Collection	sample ID	Voucher No.	Sample type	Sampling location
Nucifraga	*caryocatactes*	*rothschildi*	MFNB	Nuci_roth	-	Skin	Aksu
Nucifraga	*caryocatactes*	*rothschildi*	SNSD	Nuci_21727	MTD-C21727	Skin	Naryn
Nucifraga	*caryocatactes*	*rothschildi*	NRM	Nuci_572263	NRM572263	Skin	Chan-tengri
Nucifraga	*columbiana*		RAM	Nuci_col	Z17.8.23	Skin	Kootenay Plains
Nucifraga	*caryocatactes*	*hemispila*	SNSD	Nuci_25740	MTD-C25740	Skin	Himalaya
Nucifraga	*caryocatactes*	*hemispila*	NRM	Nuci_572264	NRM572264	Skin	N/A
Nucifraga	*caryocatactes*	*interdicta*	SNSD	Nuci_23063	MTD-C23063	Skin	Beijing
Nucifraga	*caryocatactes*	*interdicta*	NRM	Nuci_572237	NRM572237	Skin	Shansi
Nucifraga	*caryocatactes*	*macella*	ZMMU	Nuci_2155	EAK 416	Tissue	Gansu
Nucifraga	*caryocatactes*	*macella*	Collection J. Martens at SNSD	Nuci_9308	MAR 9308	Tissue	Gansu
Nucifraga	*caryocatactes*	*owstoni*	SNSD	Nuci_44762	MTD-C44762	Skin	Mt Arisa
Nucifraga	*caryocatactes*	*owstoni*	NRM	Nuci_572238	NRM572238	Skin	Taiwan
Nucifraga	*multipunctata*		SNSD	Nuci_19464	MTD-C19464	Skin	Kashmir
Nucifraga	*multipunctata*		NRM	Nuci_572239	NRM572239	Skin	Muski
Nucifraga	*multipunctata*		NHMD	Nuci_A	68845	Skin	Nuristan
Nucifraga	*multipunctata*		NHMD	Nuci_B	68855	Skin	Nuristan

Supplementary Table 8. Short read mapping statistics.

Sample ID	Species	subspecies	Number of reads (millions)	% mapped	Coverage	≥ 10X
Nuci_0516	*N. caryocatactes*	*caryocatactes*	200.5	93.50%	26.5X	85.50%
Nuci_191	*N. caryocatactes*	*caryocatactes*	165	86.60%	19.0X	80.00%
Nuci_192	*N. caryocatactes*	*caryocatactes*	187.9	94.30%	24.0X	82.50%
Nuci_S2	*N. caryocatactes*	*caryocatactes*	139.9	93.90%	17.0X	78.40%
Nuci_1600	*N. caryocatactes*	*japonica*	164.3	94.20%	17.0X	76.00%
Nuci_4353	*N. caryocatactes*	*japonica*	91.6	95.40%	10.0X	51.60%
Nuci_0754	*N. caryocatactes*	*japonica*	124.3	90.30%	15.5X	83.70%
Nuci_123	*N. caryocatactes*	*macrorhynchos*	164.3	96.30%	19.0X	77.30%
Nuci_1356	*N. caryocatactes*	*macrorhynchos*	144.45	93.20%	19X	84.40%
Nuci_2156	*N. caryocatactes*	*macrorhynchos*	179.55	91.50%	23X	85.10%
Nuci_2161	*N. caryocatactes*	*macrorhynchos*	133.3	94.50%	17.5X	84.10%
Nuci_3573	*N. caryocatactes*	*macrorhynchos*	150	92.20%	19.5X	84.50%
Nuci_N11	*N. caryocatactes*	*macrorhynchos*	173.5	95.20%	22.0X	82.00%
Nuci_122	*N. caryocatactes*	*macrorhynchos*	150.4	95.70%	20.0X	82.00%
Nuci_21727	*N. caryocatactes*	*rothschildi*	124.6	94.70%	14.0X	74.40%
Nuci_572263	*N. caryocatactes*	*rothschildi*	106.8	95.00%	14.0X	74.50%
Nuci_roth	*N. caryocatactes*	*rothschildi*	136.1	94.80%	15.0X	76.90%
Nuci_col	*N. columbiana*		120.1	94.10%	15.0X	77.40%
Nuci_25740	*N. caryocatactes*	*hemispila*	103.4	94.90%	12.0X	67.30%
Nuci_572264	*N. caryocatactes*	*hemispila*	77.2	95.40%	8.0X	40.60%
Nuci_23063	*N. caryocatactes*	*interdicta*	96.2	94.10%	10.0X	52.70%
Nuci_572237	*N. caryocatactes*	*interdicta*	123.5	86.70%	17.0X	78.30%
Nuci_2155	*N. caryocatactes*	*macella*	155.55	90.40%	20X	84.50%
Nuci_9308	*N. caryocatactes*	*macella*	158.35	92.80%	20.5X	85.10%
Nuci_44762	*N. caryocatactes*	*owstoni*	129.6	93.80%	14.0X	70.80%
Nuci_572238	*N. caryocatactes*	*owstoni*	152.3	95.20%	22.0X	81.50%
Nuci_19464	*N. multipunctata*		127.1	94.30%	13.0X	70.30%
Nuci_572239	*N. multipunctata*		154	96.20%	23.0X	81.20%
Nuci_A	*N. multipunctata*		61.8	95.90%	8.0X	24.20%
Nuci_B	*N. multipunctata*		123.7	95.20%	15.0X	74.70%
Corvus_cor	*Corvus cornix*		174.47	95.45%	15.1X	82.22%

Supplementary Table 9. Additional taxa included in the divergence times estimation. Whole mitochondrial genomes were extracted from the NCBI database for a total of 27 additional taxa in order to include selected fossil calibration points for optimal divergence time estimation.

Species	Scientific name	NCBI Acc. Number
New Zealand rock wren	*Xenicus gilviventris*	KX369033
Rifleman	*Acanthisitta chloris*	AY325307
Northern crested caracara	*Caracara cheriway*	MN231451
Kakapo	*Strigops habroptila isolate Jane*	CM013787
Kea	*Nestor notabilis*	KX369037
Greater vasa parrot	*Coracopsis vasa*	KM611468
Peregrine falcon	*Falco peregrinus*	JQ282801
Blackish-grey antshrike	*Thamnophilus nigrocinereus*	KJ909192
Great-billed seed finch	*Sporophila maximiliani*	MF327582
Tristram's bunting	*Emberiza tristrami*	HQ896035
Eurasian Magpie	*Pica pica*	HQ915867
Eurasian Jay	*Garrulus glandarius*	JN018413
Henderson's ground jay	*Podoces hendersoni*	GU592504
Azure-winged magpie	*Cyanopica cyanus koreensis*	KT934323
Azure-winged magpie	*Cyanopica cyanus*	JN108020
Alpine chough	*Pyrrhocorax graculus*	KJ598623
Red-billed chough	*Pyrrhocorax pyrrhocorax*	KJ598622
Red-billed blue magpie	*Urocissa erythrorhyncha*	JQ423932
Taiwan blue magpie	*Urocissa caerulea*	MG932654
Dusky woodswallow	*Artamus cyanopterus*	KY994613
Black-faced woodswallow	*Artamus cinereus*	MF784400
Black-naped oriole	*Oriolus chinensis*	JQ083495
Grey-cheeked fulvetta	*Alcippe morrisonia hueti*	KX376475
Fairy pitta	*Pitta nympha*	KJ680302
Ochre-bellied flycatcher	*Mionectes oleagineus*	KJ742591
Mongolian lark	*Melanocorypha mongolica*	KY887027
Hooded Crow	*Corvus cornix*	CM002877
Clark’s nutcracker	*Nucifraga columbiana*	KF509923

Supplementary Table 10. Phenotypic traits measured for the morphometric analyses. Description of morphological traits measured on museum specimens.

**Functional association**	**Morphological traits**	**Description**
Foraging and diet	Bill length	Distance from commissural point of upper and lower bill to tip of closed bill
	Bill depth	Maximum height from the angle at front of skull
	Bill width	External distance between the two commissural points
Flight ability	Wing length	Distance from carpal joint to longest primary
	Kipp's distance	Distance between tip of first secondary and tip of longest primary in naturally folded wing
	Distal distance	Distance between tip of outermost primary and tip of wing
Maneuverability	Tail length	Distance from where the bases of the two innermost rectrices emerge from the skin to the tip of the longest tail feather in naturally folded tail
	Tail graduation	Distance from tip of longest to tip of shortest tail feather in naturally folded tail
Bipedal locomotion	Back toe length	Distance between back toes and tarsus
	Tarsus length	Distance from joint between tarsus toes to intertarsal joint
	Lateral tarsus diameter	Diameter of lateral tarsus
Plumage color	White extent of tail	White extensions on the outermost rectrices measured from where the bases of the rectrices emerge from the skin

Supplementary Table 11. Plant-bird associations. References for interaction between food plants and nutcracker along with the reference for the plant distribution. For distribution maps see Supplementary Figure 1.

Scientific name	Common Name	Plant Distribution reference	Literature reference Bird association
Picea abies	Norway Spruce	Euforgen[10]	Haffer[18]; Cramp et al.[19]
Picea schrenchiana	Schrenk's Spruce	Vidakovic[11]	Grote[20,21]
Corylus avellana	Common Hazel	Bassil et al.[12]	Kleinschmidt[22]; Rolando[23]
C. chinensis	Chinese Hazel	Bassil et al.[12]	
C. colurana	Turkish Hazel	Bassil et al.[12]	
C. fargesii	Farges' Hazel	Bassil et al.[12]	
C. ferox	Himalayan Hazel	Bassil et al.[12]	
C. heterophylla	Asian Hazel	Bassil et al.[12]	
C. jacquemontii	Jacquemont's Hazel	Bassil et al.[12]	
C. sieboldiana	Japanese Hazel	Bassil et al.[12]	
Pinus cembra	Swiss stone pine	Euforgen[13]	Mattes[24,25]
P. sibirica	Siberian pine	Nakazawa et al.[14]	Grote[21]; Mezhennyj[26]
P. pumila	Siberian dwarf pine	Critchfield & Little[15]	Chiba[27]
P. koraiensis	Korean pine	Critchfield & Little[15]	Mattes[24]
P. gerardiana	Gerrard's Pine	Critchfield & Little[15]	Ali & Ripley[28]
P. parviflora	Japanese white pine	Critchfield & Little[15]	Hayashida[29,30]
P. mugo	Dwarf Mountain Pine	Critchfield & Little, [15]	Haffer, 1993[18]
P. sylvestris	Scots Pine	Nakazawa et al.[14]	Haffer, 1993[18]
P. peuce	Balkan Pine	Critchfield & Little[15]	Cramp et al.[19]
P. heldreichii	Heldreich's Pine	Critchfield & Little, [15]	Kleinschmidt[22]
P. wallichiana	Himalayan white pine	Critchfield & Little, [15]	Ali & Ripley[28]
P. nigra	Austrian Pine	Critchfield & Little[15]	Cramp et al.[19]
P. amamiana	Amami Pine	Kanetani et al.[16]	Farjón[31]
P. armandii	Armand's Pine	Critchfield & Little[15]	Farjón[31]
P. pinea	Stone Pine	Euforgen, 2009[17]	Farjón[31]
P. fenzeliana	Fenzel's Pine	Critchfield & Little[15]	Farjón[31]

References

1. Nadachowska-Brzyska, K., Burri, R., Smeds, L. & Ellegren, H. PSMC analysis of effective population sizes in molecular ecology and its application to black-and-white *Ficedula* flycatchers. *Mol. Ecol.* **25**, 1058-1072 (2016).

2. Vijay N. *et al*. Evolution of heterogeneous genome differentiation across multiple contact zones in a crow species complex. *Nat. Commun*. **7**, 13195 (2016).

3. BirdLife International Species factsheet: *Nucifraga caryocatactes*. http://www.birdlife.org (2020).

4. Päckert, M. *et al*. A revised phylogeny of nuthatches (Aves, Passeriformes, *Sitta*) reveals insight in intra- and interspecific diversification patterns in the Palearctic. *Vertebr. Zool*. **70**, 241-262 (2020).

5. Päckert, M. *et al*. Horizontal and elevational phylogeographic patterns of Himalayan and Southeast Asian forest passerines (Aves: Passeriformes). *J Biogeogr.* **39**, 556-573 (2012).

6. Päckert, M. *et al*. Out of Tibet and out of Himalayas: centers of origin and diversification of passerine birds in the Qinghai-Tibet Plateau region. *Ecol. Evol*. **10**, 9283-9300 (2020).

7. Alström, P. *et al*. Complete species-level phylogeny of the leaf warbler (Aves: Phylloscopidae) radiation. *Mol. Phylogenet. Evol.* **126**, 141-152 (2018).

8. Oliveros, C. H. *et al*. Earth history and the passerine superradiation. *Proc. Natl. Acad. Sci. U. S. A.* **116**, 7916-7925 (2019).

9. Jarvis, E. *et al*. Whole-genome analyses resolve early branches in the tree of life of modern birds. *Science* **346**, 1320–1331. (2014).

10. Euforgen, 2009. Distribution map of Norway spruce (*Picea abies*). Retrieved from www.euforgen.org on 18th October 2017.

11. Vidakovic, M. *Conifers: morphology and variation*. (Graficki zavod Hrvatske, 1991).

12. Bassil, N., Boccacci, P., Roberto, B., Postman, J., & Mehlenbacher, S. Nuclear and chloroplast microsatellite markers to assess genetic diversity and evolution in hazelnut species, hybrids and cultivars. *Genetic Resources and Crop Evolution* **60**, 543-568 (2013).

13. Euforgen, 2009. Distribution map of Swiss stone pine (*Pinus cembra*). Retrieved from www.euforgen.org on 18th October 2017.

14. Nakazawa, F. *et al*. DNA analysis for section identification of individual *Pinus* pollen grains from Belukha glacier, Altai Mountains, Russia. *Environmental Research Letters* **8**, 014032 (2013).

15. Critchfield, W. B. & Little, E. L. Geographic distribution of the pines of the world. U.S. Department of Agriculture, Washington, D.C., Miscellaneous Publication 991,1-97 (1966).

16. Kanetani, S., Kawahara, T., Kanazashi, A. & Yoshimaru, H. Diversity and conservation of genetic resources of an endangered five-needle pine species, *Pinus armandii Franch. var. amamiana* (Koidz.) Hatusima. *SDA Forest Service Proceedings*, **RMRS-P-32**, 188–191 (2004).

17. Euforgen, 2009. Distribution map of the Italian stone pine (*Pinus pinea*). Downloaded from www.euforgen.org on 18th October 2017.

18. Haffer, J. *Nucifraca caryocatactes (Linnaeus 1758) – Tannenhäher. In: Glutz von Blotzheim, U. N., Bauer, K.: Handbuch der Vogel Mitteleuropas. Vol. 13/III: Corvidae – Sturnidae*. (AULA-Verlag, 1993)

19. Cramp, S., Perrins, C. M., Brooks, D. J. *Handbook of the Birds of Europe, the Middle East and North Africa. The Birds of the Western Palearctic. Vol. VIII: Crows to Finches*. (Oxford University Press, 1994).

20. Grote, H. Zur Biologie des Tannenhähers *Nucifraga caryocatactes*. *Der Vogelzug* **11**, 127-129 (1940).

21. Grote, H. Ueber die Lebensweise des Schlankschnäbligen Tannenhähers in Sibirien. *Ornithologischer Beobachter* **44**, 84-90 (1947).

22. Kleinschmidt, O. *Corvus Nucifraga*. (Berajah, Zoographica infinita, 1909-11).

23. Rolando, A. Home range and habitat selection by the Nutcracker *Nucifraga caryocatactes* during autumn in the Alps. *Ibis* **138**, 383-390 (1996).

24. Mattes, H. Der Tannenhäher im Engadin. Studien zu seiner Ökologie und Funktion im Arvenwald. *Münsterische Geographische Arbeiten* **2**, 1-78 (1978).

25. Mattes, H. Die Lebensgemeinschaft von Tannenhäher, *Nucifraga caryocatactes* (L) und Arve, *Pinus cembra* L., und ihre forstliche Bedeutung in der oberen Gebirgswaldstufe. Eidgenössische Anstalt für das Forstliche Versuchswesen, Birmensdorf, 241, 1-74 (1982).

26. Mezhennyj, A. A. Biology of the nutcracker *Nucifraga caryocatactes macrorhynchos* in South Yakutia. *Zoologicheskii Zhurnal* **43**, 1679-1687 (1964).

27. Chiba, S. Analysis of stomach content of *Nucifraga caryocatactes* and *Apus pacificus* of Japan. *Journal of the Yamashina Institute for Ornithology* **5**, 287-297 (1968).

28. Ali, S. & Ripley, S. D. *Handbook of the Birds of India and Pakistan, together with those of Nepal, Bhutan and Ceylon. Vol. V.* (Oxford University Press, 1972).

29. Hayashida, M. Seed dispersal and regeneration patterns of *Pinus parviflora var. pentaphylla* on Mt. Apoi in Hokkaido. *Research Bulletin of Hokkaido University Forests* **46**, 177–190 (1989).

30. Hayashida, M. Role of nutcrackers on seed dispersal and establishment of *Pinus pumila* and *P. entaphylla*. In: Schmidt, W. C., Holtmeier, F. K. (eds): *Proceedings-International Workshop on Subalpine Stone Pines and Their Environment: the Status of Our Knowledge*. General Technical Report **INT-309**, Odgen: USDA Forest Service, IRS. 159–162 (1994).

31. Farjón, A. *A Handbook of the World's Conifers. Vol II.* (Brill, 2010)
