# Supplementary material for: Speciation and population divergence in a mutualistic seed dispersing bird
Source: Commun Biol. 2022 May 9;5:429. doi: 10.1038/s42003-022-03364-2 (PMC9085801; doi:10.1038/s42003-022-03364-2)
Supplement: Supplementary file 2 — Reporting Summary [file 42003_2022_3364_MOESM2_ESM.pdf]

nature research

Corresponding author(s): Dr. Maria A. Nilsson

Last updated by author(s): Jan 21, 2022

Reporting Summary

Nature Research wishes to improve the reproducibility of the work that we publish. This form provides structure for consistency and transparency in reporting. For further information on Nature Research policies, see our Editorial Policies and the Editorial Policy Checklist.

Statistics

For all statistical analyses, confirm that the following items are present in the figure legend, table legend, main text, or Methods section.

n/a	Confirmed	
☐	☒	The exact sample size (*n*) for each experimental group/condition, given as a discrete number and unit of measurement
☒	☐	A statement on whether measurements were taken from distinct samples or whether the same sample was measured repeatedly
☐	☒	The statistical test(s) used AND whether they are one- or two-sided<br>*Only common tests should be described solely by name; describe more complex techniques in the Methods section.*
☐	☒	A description of all covariates tested
☐	☒	A description of any assumptions or corrections, such as tests of normality and adjustment for multiple comparisons
☐	☒	A full description of the statistical parameters including central tendency (e.g. means) or other basic estimates (e.g. regression coefficient) AND variation (e.g. standard deviation) or associated estimates of uncertainty (e.g. confidence intervals)
☒	☐	For null hypothesis testing, the test statistic (e.g. *F*, *t*, *r*) with confidence intervals, effect sizes, degrees of freedom and *P* value noted<br>*Give P values as exact values whenever suitable.*
☐	☒	For Bayesian analysis, information on the choice of priors and Markov chain Monte Carlo settings
☒	☐	For hierarchical and complex designs, identification of the appropriate level for tests and full reporting of outcomes
☒	☐	Estimates of effect sizes (e.g. Cohen's *d*, Pearson's *r*), indicating how they were calculated

Our web collection on statistics for biologists contains articles on many of the points above.

Software and code

Policy information about availability of computer code

Data collection

No software was used

Data analysis

De novo genome assembly:
Quality control of raw read data was performed with FastQC v0.11.7 (Andrews 2010). De novo genome assembly of 10x genomics linked-reads was done using Supernova v.1.0.0 (Weisenfeld et al 2017). The quality of the assembly was assessed with QUAST v. 6.4.3 (Gurevich et al., 2013) and gene completeness was assessed with BUSCO v.3.0.2 (Seppey et al 2019). Synteny analyses was done with JupiterPlot (https://github.com/JustinChu/JupiterPlot). RepeatModeler v.1.0.11 (Smit & Hubley 2015) was used to identify de novo repeats in the nutcracker genome. Subsequent repeatmasking was performed with RepeatMasker v.4.1.0 (Smit et al., 2013).

Re-sequencing and mapping:
Re-sequencing data was analyzed using a specific pipeline designed for handling sequencing data from museum specimen according to a cleaning workflow available at https://github.com/mozesblom. It included quality control with FastQC v0.11.7, deduplication with Super-Deduper v2.0 (https://github.com/dstreett/Super-Deduper), trimming with Trimmomatic v.0.38 (Bolger et al., 2014), merging of overlapping reads with PEAR v0.9.6 (Zhang et al., 2014) and removal of low complexity reads. Mapping of reads was performed with BWA mem v.0.7.17 (Li & Durbin 2009). Samfiles were converted to bamfiles and sorted with samtools v 1.7 (Li et al., 2009). Duplicates were marked with Picard v.2.18.21 (http://broadinstitute.github.io/picard/). Freebayes v1.3.1 (Garrison & Marth 2012) was used to perform variant calling.

Phylogenetic analyses:
The consensus function of Bcftools v.1.2 (Li et al., 2009) was used to call concensus sequences. Alignment of mitochondrial sequences was performed with MAFFT v7.407 (Nakamura et al., 2018) and subsequent maximum likelihood analyses was performed with IQtree v.1.6.11 (Nguyen et al., 2014). Phylogenomic inference of the nuclear genome consisted of removal of heterozygous sites and N's in the consensus sequences for all individuals using bedtools v2.28.0. Consensus sequences were subsequently aligned using custom designed scripts available at https://github.com/mobilegenome/phylogenomics/. Maximum likelihood inference and approximate unbiased (AU)-tests were performed

with IQtree v.1.6.11, whereas species tree inference based on the multispecies coalescent (MSC) was performed with ASTRAL-III v5.6.1 (Zhang et al., 2018). Network analyses was performed using SplitsTree v.4.15.1 (Huson & Bryant 2006).

Allele-based analyses:
VCF files were filtered with VCFtools v 0.1.17. (Danecek et al 2011) for biallelic positions and a minimum and maximum coverage ranging from 8X to 2.5 times mean depth of the respective sample. To account for the bias resulting from mapping to an in-group reference genome, we applied a strict filtering approach in which at maximum one individual was allowed to have missing data at each site. VCF-files were converted to plink's PED format with VCFtools v 0.1.17 and principal component analyses was subsequently performed with Plink version 1.9 (Purcell 2007). To account for linkage disequilibrium, sites within a 50-SNP stepping window with a correlation coefficient higher than 0.1 were omitted. Additionally, ADMIXTURE v1.3.0 (Alexander & Lange 2011) was used to estimate population structure within the data with K ranging from 2 to 4 for both between- and within species. ADMIXTURE's cross validation was used to estimate the most likely number of K.

Divergence time estimation:
Divergence times were estimated using BEAST2 v. 2.7.0 (Bouckaert et al 2019). Transition-transversion split was used to find a fitting substitution model. Zero offset values were based on the minimum fossil ages and the log mean and standard deviation were adjusted to achieve the optimal match of the lognormal prior distribution for the fossil age interval. All calibrated nodes were set to be monophyletic. BEAST was run for 100,000,000 MCMC iterations with a burn-in of 10% for the calibrated tree.

Reconstruction of historical effective population size:
Historical effective population size was reconstructed using the Pairwise Sequentially Markovian Coalescent (PSMC) model as implemented in PSMC v.0.6.5 (Li & Durbin 2011). Whole-genome diploid consensus sequences were created with the 'mpileup' command of Samtools v 1.7. and the "view" option of BCFtools v.1.9 (https://samtools.github.io/bcftools/) in combination with the included script 'vcfutils.pl'. Sites with a read depth below 8 or above twice the sample's median depth and a minimum mapping quality below 20 were removed. PSMC was run for 25 iterations with the upper limit of the TMRCA set to -t 5, the initial h/q value to -r 1, and 34 atomic time intervals (4+30*2+4+6+10). Bootstrapping was performed for 100 iterations and results were scaled with a mutation rate (m) of 3.18 × 10–9 substitutions per generation and a generation time of 7 years.

Morphometric analyses:
Linear discriminant analyses of morphometric traits was performed with the ggord package (Beck 2017) in R v. 3.5.2. (R Core Team 2020). Statistical testing of comparisons between groups was performed with Welch's t-test in R v. 3.5.2.

For manuscripts utilizing custom algorithms or software that are central to the research but not yet described in published literature, software must be made available to editors and reviewers. We strongly encourage code deposition in a community repository (e.g. GitHub). See the Nature Research guidelines for submitting code & software for further information.

Data

Policy information about availability of data

All manuscripts must include a data availability statement. This statement should provide the following information, where applicable:

- Accession codes, unique identifiers, or web links for publicly available datasets
- A list of figures that have associated raw data
- A description of any restrictions on data availability

The data produced in the current study are available at the NCBI Sequence Read Archive under BioProject PRJNA682958 accession numbers SAMN17014695–SAMN17014724. The raw 10X genomics sequencing data used for the nutcracker reference assembly has been deposited at NCBI under accession number SAMN17050349.

Field-specific reporting

Please select the one below that is the best fit for your research. If you are not sure, read the appropriate sections before making your selection.

☐ Life sciences ☐ Behavioural & social sciences ☒ Ecological, evolutionary & environmental sciences

For a reference copy of the document with all sections, see nature.com/documents/nr-reporting-summary-flat.pdf

Ecological, evolutionary & environmental sciences study design

All studies must disclose on these points even when the disclosure is negative.

Study description	In the current study we examined whether the evolution of Eurasian nutcrackers was driven by its mutualism with pines. We included whole genome resequencing data of 31 individuals covering the complete nutcracker distribution range. Additionally, we used an independent morphometric dataset based on the measurements of 118 museum specimens to assess the phenotypic differentiation between nutcrackers.
Research sample	A total of 31 individuals were included in the study in order to get a full scope of the evolutionary history of this species complex. Samples covered both Eurasian nutcracker species (N. caryocatactes and N. multipunctata). Additionally, one specimen of the Clark's nutcracker (N. columbiana) was included as an outgroup for the analyses.
Sampling strategy	Sampling strategy was based on including at least a minimum of two individuals per subspecies in order to have a representation of each subspecies for the phylogenomic analyses. Specimen were chosen to maximize the geographic range for each subspecies.
Data collection	DNA extraction was either performed at the corresponding sequencing company or done at the Senckenberg lab by J. de Raad.

Data collection	Subsequent sequencing data was collected and organized by J. de Raad. Morphometric analyses of museum specimens was done by E. Neuschulz, M. Schleuning and T. Töpfer.
Timing and spatial scale	N/A
Data exclusions	Of the 118 museum specimens measured for the morphometric analyses, 28 had to be excluded due to missing data or inproper/ incomplete measurements.
Reproducibility	All the raw data used in the study are available at the NCBI database
Randomization	Samples were grouped based on their original specimen labels and collection site.
Blinding	N/A

Did the study involve field work? ☐ Yes ☒ No

Reporting for specific materials, systems and methods

We require information from authors about some types of materials, experimental systems and methods used in many studies. Here, indicate whether each material, system or method listed is relevant to your study. If you are not sure if a list item applies to your research, read the appropriate section before selecting a response.

Materials & experimental systems

n/a	Involved in the study	
☒	☐	Antibodies
☒	☐	Eukaryotic cell lines
☒	☐	Palaeontology and archaeology
☐	☒	Animals and other organisms
☒	☐	Human research participants
☒	☐	Clinical data
☒	☐	Dual use research of concern

Methods

n/a	Involved in the study	
☒	☐	ChIP-seq
☒	☐	Flow cytometry
☒	☐	MRI-based neuroimaging

Animals and other organisms

Policy information about studies involving animals; ARRIVE guidelines recommended for reporting animal research

Laboratory animals	N/A
Wild animals	N/A
Field-collected samples	N/A
Ethics oversight	No ethical approval was needed. Where required, approval of the Nagoya regulations was arranged.

Note that full information on the approval of the study protocol must also be provided in the manuscript.
